# Dynamic covalent chemistry in live cells for organelle targeting and enhanced photodynamic action†

**DOI:** 10.1039/d1sc04770a

**Published:** 2022-02-04

**Authors:** Fei Liu, Dmytro I. Danylchuk, Bohdan Andreiuk, Andrey S. Klymchenko

**Affiliations:** Laboratoire de Bioimagerie et Pathologies, UMR 7021 CNRS, ITI Chimie des Systèmes Complexes, Université de Strasbourg 74 Route du Rhin 67401 Illkirch France andrey.klymchenko@unistra.fr

## Abstract

Organelle-specific targeting enables increasing the therapeutic index of drugs and localizing probes for better visualization of cellular processes. Current targeting strategies require conjugation of a molecule of interest with organelle-targeting ligands. Here, we propose a concept of dynamic covalent targeting of organelles where the molecule is conjugated with its ligand directly inside live cells through a dynamic covalent bond. For this purpose, we prepared a series of organelle-targeting ligands with a hydrazide residue for reacting with dyes and drugs bearing a ketone group. We show that dynamic hydrazone bond can be formed between these hydrazide ligands and a ketone-functionalized Nile Red dye (NRK) *in situ* in model lipid membranes or nanoemulsion droplets. Fluorescence imaging in live cells reveals that the targeting hydrazide ligands can induce preferential localization of NRK dye and an anti-cancer drug doxorubicin in plasma membranes, mitochondria and lipid droplets. Thus, with help of the dynamic covalent targeting, it becomes possible to direct a given bioactive molecule to any desired organelle inside the cell without its initial functionalization by the targeting ligand. Localizing the same NRK dye in different organelles by the hydrazide ligands is found to affect drastically its photodynamic activity, with the most pronounced phototoxic effects in mitochondria and plasma membranes. The capacity of this approach to tune biological activity of molecules can improve efficacy of drugs and help to understand better their intracellular mechanisms.

## Abstract 2

We introduce a concept of dynamic covalent targeting of organelles, where a dye/drug molecule is conjugated with its targeting ligand inside live cells by a reversible hydrazone bond, revealing organelle-dependent photodynamic action.

## Graphical abstract

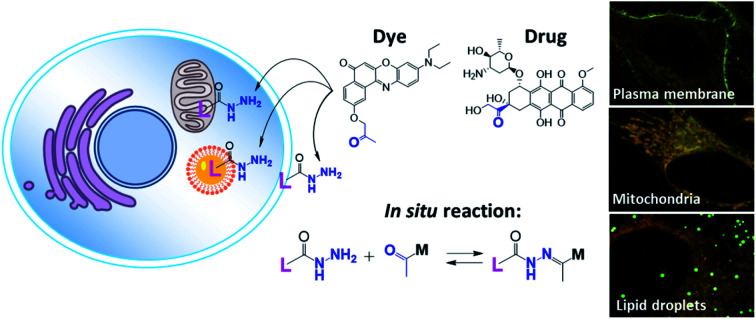

## Introduction

Organelle-specific targeting in live cells is an emerging field of bioimaging and drug delivery.^1–7^ Targeting therapeutic molecules to desired intracellular areas can improve their efficacy and help to understand mechanisms of their action.^8–11^ On the other hand, targeting fluorescent probes to specific organelles is useful for monitoring and tracking of behavior and functions of different organelles in normal and stress conditions.^2,5,12–14^ Previous studies showed that photosensitizers (PS) targeted to cell organelles, especially mitochondria and plasma membranes could improve the efficacy and precision of photodynamic therapy (PDT).^15–22^ This remarkable effect originates from the short half-life of singlet oxygen (^1^O_2_) (∼40 ns), generated locally by PS after light excitation, which could only diffuse within the limited distance from PS (∼20 nm).^23–25^

To address a specific organelle, the molecules of interest are usually conjugated with targeting units,^2,11^ well established for plasma membrane,^26^ lysosomes,^27^ mitochondria,^28,29^ and endoplasmic reticulum (ER).^30^ However, chemical modification may alter the properties of the molecule, which presents an important limitation of the method. Alternatively, specific targeting of cell compartments could be achieved using bioorthogonal chemistry,^31–33^ where a molecule of interest can react in cells with a target molecule (*e.g.* sugars or lipids), usually using highly efficient “click” reactions.^34,35^ Moreover, target proteins fused with protein tags, such as the SNAP-tag,^36^ CLIP-tag^37^ and HaloTag^38^ can react with corresponding chemical ligands, which allows targeting of small (dye) molecules to specific organelles and imaging their local properties.^39,40^ However, these approaches exploit essentially irreversible reactions, so that the molecule localized within a target organelle is chemically modified. Is it possible to target the same molecule to different specific cell compartments while preserving its native chemical structure before and after this targeting processes? One could consider dynamic covalent chemistry to localize a molecule by an *in situ* reaction with targeting ligand localized in a given cell compartment. In this case, even after targeting to the site (organelle) of interest, reversible processes will ensure presence of unmodified species. Dynamic covalent chemistry is a powerful approach to generate and break covalent bonds (including imine, hydrazone, disulfide, *etc.*) under control of environment.^41–44^ Nature uses dynamic covalent bonds (disulfide) to maintain the tertiary structure of proteins, while chemists use them for bioconjugation^45^ and to generate responsive, self-healing and adaptive materials.^46–48^ Dynamic covalent chemistry has been also applied for a controlled drug release^49^ and intracellular delivery.^50,51^ In the previous works dynamic imine bonds enabled targeting amino-lipid at the cell surface, which was used for detection of bacteria^52^ or targeting specific proteins with aldehyde derivatives of dyes.^53^ However, dynamic covalent chemistry has not been explored to date for localizing molecules in cellular organelles. For this purpose, hydrazone bonds are particularly attractive because of their relatively high stability and applicability for bioconjugation,^45^ drug and gene delivery.^54–57^ Our recent study showed that hydrazone bond could help nanoemulsions to uptake and release free ketones in the environment, like a “drug sponge”.^58^ We hypothesized that *in situ* formation of hydrazone dynamic bonds could be a driving force for localizing molecules in cell compartments, which could enable tuning their biological activity.

In the present study, we introduce a concept of “dynamic covalent targeting” of organelles based on reversible hydrazone bond formation between a molecule of interest (a dye or a drug bearing a ketone moiety) and a ligand localized in specific subcellular compartments, such as plasma membranes, lipid droplets and mitochondria. We show that dynamic covalent targeting of the same dye molecule to different organelles can tune its photodynamic action in cells.

## Results and discussion

**Scheme 1:**
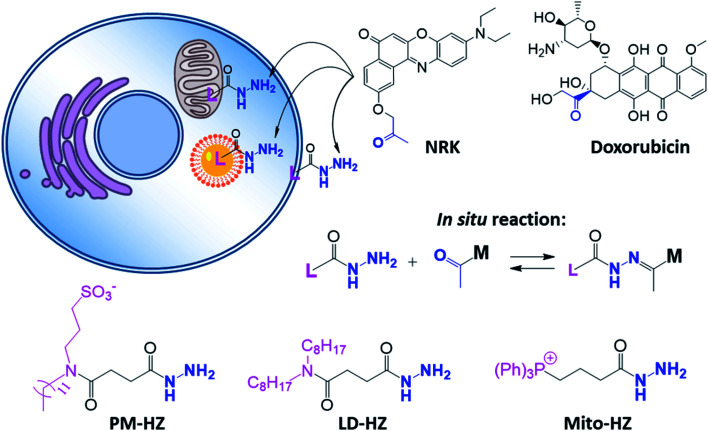
Hydrazide derivatives for dynamic covalent targeting of organelles with Nile Red-ketone (NRK) dye and doxorubicin drug.

In our concept of dynamic covalent targeting, a specially designed targeting ligand, bearing hydrazide reactive group will be localized in the cell organelle of interest and then form *in situ* a dynamic covalent bond with a given molecule bearing a ketone group. In this case, a ketone molecule, which does not have any organelle specificity, may redistribute due to the *in situ* reaction and thus localize in the targeted organelle (Scheme 1). In this case, the ketone molecule does not require any pre-functionalization with the targeting ligand. Then, after *in situ* reaction with the target site, dynamic nature of the hydrazone bond will ensure reversibility of the process, allowing presence of free ketone molecules (Scheme 1). In order to direct ketone containing Nile Red dye NRK (Scheme 1) into different cell organelles, we designed three hydrazide bearing ligands. Triphenylphosphonium was used to achieve mitochondria targeting due to its ability to accumulate in mitochondria driven by their transmembrane potential.^2,7,29^ To target lipid droplets (LDs), a hydrazide moiety was functionalized with two octyl chains in order to favor partitioning of molecules into hydrophobic oily core of LDs.^59,60^ For plasma membrane targeting, we used an anchor group, composed of dodecyl chain and sulfonate, which was recently proposed for designing membrane probes.^26^ The targeting groups were conjugated to the hydrazide using short linkers, based on either succinic acid or butyric acid (Scheme 1). As ketone molecules, we selected a fluorescent dye NRK, composed of Nile Red dye and ketone moiety,^58^ and an anti-cancer drug doxorubicin.^61,62^ The Nile Red fluorophore, owing to the solvatochromic and fluorogenic properties,^63^ is expected to light up and blue shift its emission after binding to lipid rich compartments.^12,26,63^ Moreover, previous study showed that NRK stained indiscriminately different lipid compartments of the cells,^58^ which is suitable to test our dynamic targeting concept. On the other hand, doxorubicin was selected because this common anti-cancer drug is fluorescent and it originally bears the ketone moiety.

**Fig. 1:**
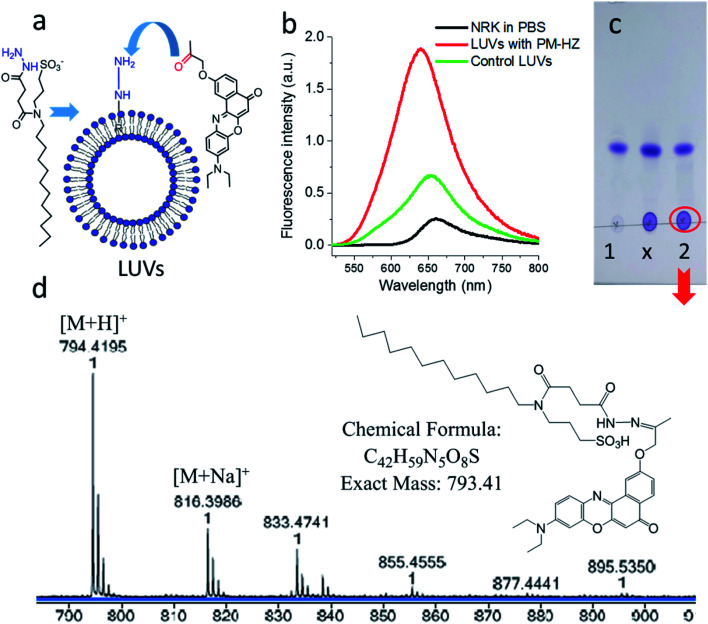
Studies of *in situ* reaction of NRK with targeting hydrazide PM-HZ in LUVs composed of DOPC. PM-HZ (50 μM) was pre-incubated for 30 min with LUVs (1 mM), dialyzed to remove excess of PM-HZ and then NRK (15 μM) was added. (a) Scheme of a model reaction of NRK with PM-HZ in LUVs. (b) Fluorescence spectra of NRK (2 μM) in PBS, blank LUVs (10 μM) and dialyzed LUVs (10 μM) with PM-HZ. Excitation wavelength was 480 nm. (c) TLC result (DCM : MeOH = 95 : 5 as an eluent) of DCM extraction of NRK in blank LUVs and LUVs containing PM-HZ: 1 – control sample (with blank LUVs), *x* – cross point, 2 – reaction. (d) Mass identification of red circled point in (c).

Firstly, the *in situ* reaction of NRK with plasma membrane targeting hydrazide (PM-HZ) was verified in model lipid membranes, large unilamellar vesicles (LUVs) composed of dioleoylphosphatidylcholine (DOPC, Fig. 1a). In a buffer, NRK showed expected weak red-shifted fluorescence, whereas in the presence of LUVs containing PM-HZ, a strong increase in the fluorescence intensity was observed together with a blue shift (Fig. 1b). Without the targeting unit (Fig. 1b), NRK showed much smaller fluorescence enhancement and blue shift, suggesting low affinity of NRK to lipid membranes. Thus, PM-HZ favors binding of NRK to lipid membranes. To identify the formation of the hydrazone conjugate, the mixtures of NRK with LUVs containing PM-HZ were subjected to extraction with dichloromethane, followed by thin layer chromatography (TLC) and mass spectrometry analysis. According to the TLC (Fig. 1c), in the presence of PM-HZ, a new spot with small *R*_f_ value appeared, that could be assigned to a hydrazone conjugate containing highly polar and charged sulfonate group. The mass spectrum of this new TLC spot confirmed the presence of the desired conjugate (Fig. 1d). These results provide an evidence for the hydrazone formation between PM-HZ and NRK *in situ* at the lipid membranes. Chromatographic separation and further spectrophotometric analysis revealed that the extract from the reaction mixture in LUVs contained 65 mol% of the conjugate (Fig. S1†), indicating that the reaction equilibrium was shifted towards hydrazone conjugate. Similar experiments were also conducted for an *in situ* reaction of NRK with Mito-HZ in lipid vesicles, yielding 28 mol% of Mito-HZ hydrazone with NRK, which was identified by mass spectrometry (Fig. S1†). In case of LD-HZ, we performed an *in situ* reaction with NRK lipid nanoemulsions, which are oil-core/surfactant-shell nanostructures mimicking intracellular lipid droplets.^64^ It was found that the reaction mixture contained 63 mol% of LD-HZ hydrazone with NRK, which was also identified by mass spectrometry (Fig. S1†). Thus, all three targeting hydrazide ligands can form hydrazones with NRK in corresponding lipid environment of membranes and nanoemulsion droplets. This reaction is probably favored by local apolar environment and high concertation of the reactive components in the lipid nanostructures, in line with the earlier studies on the hydrazone formation in lipid nanoemulsions.^58^

**Fig. 2:**
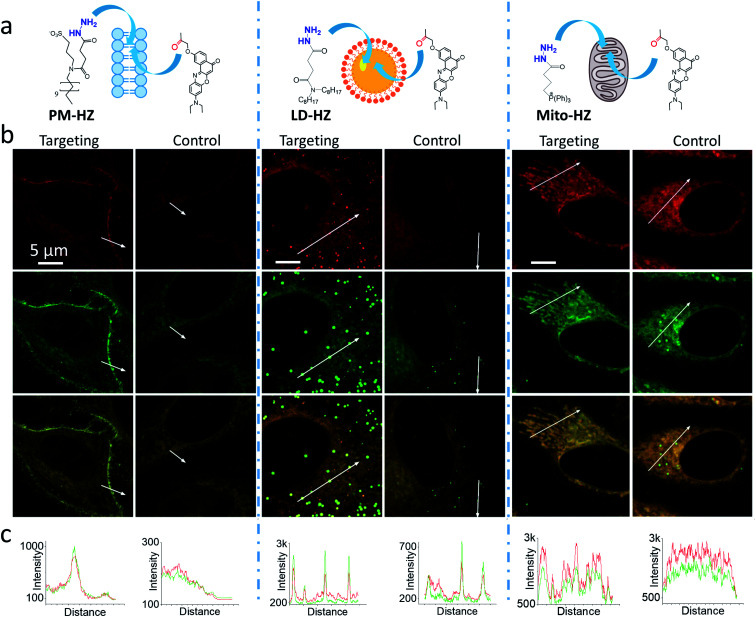
(a) Targeting NRK dye to plasma membrane, lipid droplets and mitochondria by corresponding hydrazide ligands: PM-HZ, LD-HZ and Mito-HZ. (b) Confocal images at the red channel (600–670 nm, upper panels) and the green channel (550–600 nm, middle panels) for NRK emission and the merge (lower panels); excitation: 488 nm. (c) Plot profiles for the red and green channels from white arrows indicated in images. Targeting agent (100 μM) was pre-incubated with cells for 30 min at 37 °C, followed by washing with PBS and then addition of NRK (1 μM for PM-HZ, LD-HZ and 2.5 μM for Mito-HZ) in Opti-MEM and incubation for 30 min at 37 °C. In controls, NRK was used directly without the targeting agent.

Then, we performed cellular studies in order to evaluate the capacity of PM-HZ to induce accumulation of NRK in plasma membranes. First, HeLa cells were incubated with different concentrations of PM-HZ, followed by NRK addition. Starting from 5 μM concentration of HZ-PM, a tendency of NRK accumulation in the plasma membranes was already observed (Fig. S2†). This effect was even more obvious at higher concentrations, 20 and 100 μM. In the further experiments, to remove the excess PM-HZ that could eventually react with NRK outside the cells, HeLa cells were washed after treatment with PM-HZ, and then incubated with NRK followed by confocal fluorescence microscopy. The fluorescence imaging using green and red channels was done in order to evaluate the solvatochromic response of Nile Red moiety on binding to lipid structures.^12,26,65^ In the control, NRK showed rather even distribution throughout the cell interior, in line with the previous work.^58^ In contrast, the cells pretreated with PM-HZ showed significant enhancement of their plasma membrane fluorescence in both emission channels (Fig. 2 and S3†). The effect was clearly seen on the line profile across the plasma membrane, showing a sharp pick, which was not observed in the control cells (Fig. 2c). These observations were confirmed by colocalization with a membrane probe F2N12SM,^66^ where the cell contour of the plasma membrane corresponded well to the signal from NRK in the presence of PM-HZ (Fig. S4†). Pearson's and Mander's correlation coefficient values were higher in the presence of the membrane targeting hydrazide (0.55 and 0.42, respectively) compared to control NRK (0.20 and 0.13, respectively) (Table S1†). Moreover, the fluorescence intensity ratio from plasma membrane *versus* whole cell was significantly higher (2.06) compared to the control (0.88). Thus, hydrazide PM-HZ favored redistribution the dye ketone towards plasma membrane. In the merged two-color images, these plasma membrane structures appeared more greenish compared to yellow-orange intracellular parts. The color variation was further confirmed by the line profile (Fig. 2c), where the peak of the green channel was higher compared to the red one, being similar for other cellular compartments. The observed color change corresponds to lower polarity in the plasma membrane, which was systematically observed for different solvatochromic probes.^12,67,68^

To verify the dynamic nature of our targeting approach, we first treated the cells with PM-HZ (20 μM) and NRK and then washed the cells three times to remove NRK and PM-HZ from the extracellular medium and replaced it with new medium at neutral pH (7.4) or low pH (5.8). It was observed that at neutral pH after washing, the signal from plasma membranes was still detectable (especially in the merged two-color images), but it was lower than before washing (Fig. S5†). At low pH, the effect was even stronger, so that the NRK distribution became the same as in the control without PM-HZ (Fig. S5†). Quantitative analysis of NRK co-localization with the plasma membrane marker F2N12SM (average fluorescence intensity ratio of plasma membrane *vs.* the whole cell as well as Pearson's and Mander's coefficients) confirmed the decrease in the localization of NRK in plasma membranes in the neutral pH medium after washing and the co-localization values further decreased after 1 h incubation (Table S2†). Moreover, the co-localization parameters were the lowest for the low pH (Table S2†). In all control experiments without PM-HZ, the colocalization parameters were low for all conditions, as expected (Fig. S5†). These results indicate that the targeting of NRK to plasma membranes is a reversible process. Indeed, washing out PM-HZ and NRK from the extracellular medium shifted the equilibrium toward hydrazone bond disruption, leading to NRK release into the medium and inside the cells, and, thus, decreased signal from the plasma membranes. Moreover, in the low pH medium after washing, the destabilization of the hydrazone bond led to nearly complete hydrolysis of the hydrazone and non-specific distribution of NRK within the cell, similar to the control without PM-HZ (Fig. S5†).

Next, we explored the possibility to target LDs in cells pretreated with LD-HZ. In comparison to the control cells, those incubated with LD-HZ showed strong dot-shaped fluorescence, which was observed as much brighter dots and peaks of high intensity in the line profiles (Fig. 2b and c). These dots colocalized well with the near-infrared marker of LDs SMCy5.5,^69^ which was also confirmed by the line profiles for the two dyes (Fig. S6†). For control cells, the signal from dots was much weaker, so that the colocalization with SMCy5.5 marker was less clear. The Pearson's and Mander's correlation coefficients were higher in the presence of LD-HZ (0.68 and 0.77, respectively) than that of control (0.52 and 0.26, respectively) (Table S1†). Moreover, the targeting hydrazide LD-HZ increased the ratio of mean fluorescence intensity of LDs/whole cell from 1.67 (control) to 4.46 (Table S1†). These results confirm that our system combining LD-HZ and NRK ensures selective dye accumulation in LDs, whereas for control NRK the partitioning into LDs is not efficient. The two-color images showed clear green pseudo-color of LDs, in contrast to the rest of the cytoplasm, in agreement with line profiles, where green emission was much stronger at the peaks corresponding to LDs (Fig. 2 and S6†). LDs are known to present much lower polarity of their lipid core, compared to any other lipid structures,^59,60,67,70^ which explains the dominant green emission of solvatochromic Nile Red moiety.

Finally, we tested our mitochondria-targeting hydrazide Mito-HZ in combination with NRK. In the presence of Mito-HZ, the characteristic mitochondrial staining could be identified in contrast to that of the control (Fig. 2b and S7†). The line profile across the cells revealed clear differences between control cells and those pre-treated with Mito-HZ (Fig. 2c). The observed structures colocalized with the mitochondria staining by MitoTracker Deep Red FM 644/665, in contrast to the control cells stained only with NRK (Fig. S7†). However, the targeting effect was much weaker than that for LDs or plasma membranes, according to ratio of mean fluorescence intensity in mitochondria/whole cell (1.73 with Mito-HZ *vs.* 1.58 in the control), Pearson's correlation coefficient (0.75 with Mito-HZ *vs.* 0.65 in the control) and Mander's correlation coefficient (0.44 with Mito-HZ *vs.* 0.54 in the control) (Table S1†). The weaker mitochondria targeting is in line with the lower yield of hydrazone formation observed in models for Mito-HZ compared to other two targeting hydrazide ligands (see above). Moreover, we assume that some fluorescence from other cell organelles might overlap with fluorescence from mitochondria, which complicates the co-localization analysis. Thus, all three targeting hydrazides induced selective accumulation of a ketone-functionalized dye in specific cellular organelles. This implies the formation of dynamic covalent bond (hydrazone) with the ligand through an orthogonal reaction, which can provide sufficient driving force to direct originally non-specific dye to the targeted subcellular compartment.

**Fig. 3:**
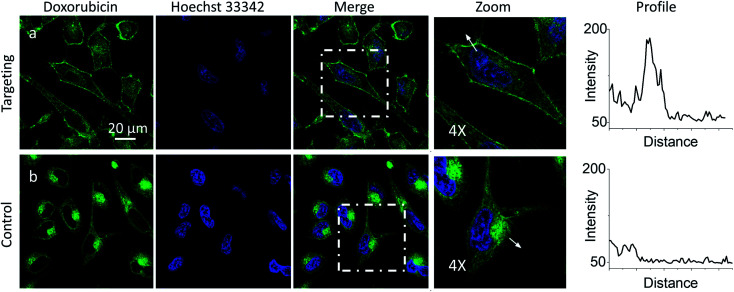
Targeting doxorubicin to plasma membranes. Cells were pre-incubated for 10 min at 37 °C with (a) or without (b) PM-HZ (20 μM) followed by addition doxorubicin (1 μM) and incubation for 30 min at 37 °C. Condition for the green channel (doxorubicin imaging): excitation at 488 nm, emission filter at 520–650 nm. Condition for the blue channel (Hoechst 33342 imaging): excitation at 405 nm, emission filter at 420–480 nm. The line profiles (right panels) of the image in the green channel is indicated by white arrows in 4× magnified images.

Then, we explored a possibility to extend our dynamic covalent targeting concept to a drug molecule bearing ketone group, such as doxorubicin. The control cells incubated with doxorubicin (for 30 min) showed fluorescence all over the cytoplasm (Fig. 3b), in line with the previous studies (nuclear localization of doxorubicin is commonly observed in cells lines poorly resistant to this drug^71^).^58,72,73^ Remarkably, in the presence of PM-HZ, the intracellular fluorescence of doxorubicin changed drastically, showing preferential localization at the level of plasma membranes (Fig. 3a), similar to dye NRK. Line profiles across the cell membrane confirmed this observation (Fig. 3). Colocalization imaging with a membrane probe F2N12SM (Fig. S8†) confirmed the membrane targeting, while the colocalization analysis based on Pearson's and Mander's coefficients as well as the intensity ratio (plasma membrane/whole cell) showed significantly improved localization in the plasma membranes compared to control cells without PM-HZ (Table S1†).

**Fig. 4:**
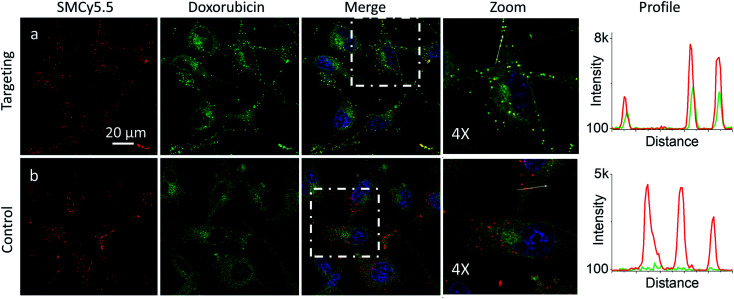
Targeting doxorubicin to lipid droplets. Cells were pre-incubated for 10 min at 37 °C with (a) or without (b) LD-HZ (20 μM) followed by addition doxorubicin (1 μM) and incubation for 30 min at 37 °C. Conditions for red channel (lipid droplet marker SMCy5.5 imaging): excitation at 644 nm, emission filter at 650–800 nm; the green channel (doxorubicin imaging): excitation at 488 nm, emission filter at 520–650 nm; the blue channel (Hoechst 33342 imaging): excitation at 405 nm, emission filter at 420–480 nm. The line profiles (right panels) of the image in the green channel is indicated by white arrows in 4× magnified images.

In the presence of LD-HZ, doxorubicin displayed strong dotted fluorescence, which colocalized well with the marker of LDs SMCy5.5 according to the two-colour images and the corresponding line profiles (Fig. 4a). Thus, doxorubicin, which does not show any affinity to LDs in control cells (Fig. 4b), can be directed towards LDs using LD-HZ. The colocalization analysis based on Pearson's and Mander's coefficients as well as the intensity ratio (organelle/whole cell) confirmed drastically improved localization of doxorubicin in the lipid droplets compared to control cells without LD-HZ (Table S1†). Likewise, the capability of Mito-HZ to direct doxorubicin towards mitochondria was also tested. In the presence of Mito-HZ the characteristic mitochondrial staining could be identified, which could be seen from the line profiles of the images of targeted doxorubicin and MitoTracker Deep Red FM 644/665 (Fig. S9†). In contrast, control cells with doxorubicin did not show a clear colocalization with mitochondria (Fig. S9†). However, one should note that mitochondria targeting was much less specific compared to the plasma membrane and LDs targeting, which was in line with the data using NRK dye. This was confirmed by only limited changes in the Pearson's and Mander's coefficients as well as the intensity ratio (mitochondria/whole cell) compared to control cells (Table S1†). Overall, these results show that our targeting hydrazides can tune intracellular localization of the ketone drug, so that dynamic covalent bonds provide a driving force for the repartitioning of the drug to the target organelle. It should be noted that it is the first time that intracellular localization of a drug can be tuned without its chemical pre-functionalization.

**Fig. 5:**
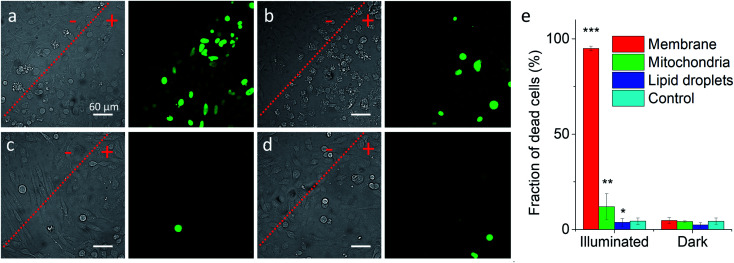
Phototoxicity of targeted NRK dye evaluated by CellTox™ Green assay. Transmission (left panels) and CellTox™ Green fluorescence (right panels) of cells pretreated with (a) PM-HZ, (b) Mito-HZ and (c) LD-HZ at and (d) control. Targeting agent (100 μM) was pre-incubated with cells for 30 min at 37 °C, followed by washing with PBS and then addition of NRK (1 μM) in Opti-MEM and incubation for 30 min at 37 °C. Areas labelled as “+” were illuminated under microscope for 1 min at 550 nm, while “−” areas were not illuminated. Images were taken after incubation for 30 min with CellTox™ Green assay. (e) Phototoxicity analysis by using Hoechst-CellTox™ Green assay co-staining (based on data shown in Fig. S11†).

Previous works showed that Nile Red derivatives can be phototoxic after continuous illumination in wide-field fluorescence microscopy.^74^ Therefore, NRK could be a suitable model to verify whether a PDT effect of the same dye can be tuned by targeting it to different organelles. To this end, cells pretreated with different targeting hydrazides were stained with NRK and then illuminated in wide-field microscopy conditions in order to induce a photodamage. It should be noted that the mean fluorescence intensity of NRK in cells was at the similar level for all studied conditions, which ensured that the amount of internalized NRK was similar in all these cases (Fig. S10†). To monitor cell death, CellTox™ Green assay was used, which stains nucleus of dead cells. In case of PM-HZ, illuminated (+) area revealed strong green fluorescence from dead cells, in contrast to non-illuminated area with practically no signs of cell damage (Fig. 5a). Similar though smaller effects were observed for Mito-HZ (Fig. 5b). In sharp contrast, only few dead cells were observed in the illuminated area of either non-targeted NRK or LD-targeted NRK (Fig. 5c and d), indicating that these conditions provided much smaller phototoxic effects. The quantitative analysis of the CellTox™ Green fluorescence (Fig. 5e) in the large population of cells (Fig. S11†) confirmed the strongest phototoxic effect for plasma membrane targeting (90% cell death) and less prominent effect for the mitochondria targeting, whereas the phototoxicity of non-targeted and LD-targeted NRK was negligible. In the bright-field images, detaching round cells were clearly observed for the membrane- and mitochondria-targeted NRK, whereas for control and LD-targeted NRK, the cell morphology did not change after the illumination.

In the time-lapse bright-field images, gradual morphology changes were observed only for cells with NRK targeted to plasma membrane and mitochondria (Fig. S12†): a formation of large membrane blebs together with gradual cell detachment in the former case and dense small bubbles at the surface of cells in the latter case. These morphological alternations indicate the cell damage, in line with the CellTox™ Green assay. Overall, these results show that phototoxic effects at the level of plasma membrane and mitochondria are much more important than at other membrane regions of the cells, in line with previous studies.^15,18–21,75^ Moreover, phototoxic damage in cell plasma membranes and mitochondria leads to different morphological changes of the cells, which shows that the phototoxicity profile can be finely tuned by the subcellular localization of the photosensitizer dye.

## Conclusions

In conclusion, a concept of “dynamic covalent targeting” of organelles is proposed based on reversible covalent bond (hydrazone) formation in live cells, which allows tuning subcellular localization of the same molecule (a dye or a drug) and reveals organelle-dependent phototoxicity. Three hydrazides bearing organelle-targeting moieties have been developed, which enable localizing Nile Red-ketone dye and anti-cancer drug doxorubicin specifically in plasma membranes, mitochondria and LDs of live cells. To the best of our knowledge, this is the first method capable to target an unmodified drug molecule to any desired location inside the cells. It is important to note that the dynamic covalent targeting works on two molecules of very different chemical structure (NRK and doxorubicin), which shows that the concept is rather universal for targeting ketone molecules. We also found that the same photosensitizer dye targeted to plasma membranes and mitochondria produced much stronger phototoxic effect compared to non-targeted or LDs-targeted dye. Thus, by localizing dye molecules in desired subcellular compartments, our approach provides fine tuning the dye phototoxicity, important for effective and precise photodynamic therapy. Moreover, capacity of the dynamic covalent targeting approach to provide specific localization to some drugs (*e.g.* doxorubicin) could enable optimizing their efficacy and better understanding their intracellular mechanisms. We should stress that reversible nature of dynamic covalent chemistry provides unique opportunities in the field of targeted drug delivery, where a non-functionalized drug can be localized in the target organelle and further released in a free form due to the dynamic equilibrium. Finally, we expect that this concept could be extended to other biologically active molecules and dynamic covalent bonds.

## Data availability

The datasets supporting this article have been uploaded as part of the ESI.†

## Author contributions

ASK proposed the concept and supervised the project. ASK and FL designed the experiments. FL performed and analyzed most of the experiments. DID, FL, and BA synthesized new molecules. ASK and FL wrote the manuscript. All authors contributed to preparation of the manuscript.

## Conflicts of interest

The authors declare no competing financial interest.

## Supplementary Material

SC-013-D1SC04770A-s001
