# Supplementary material for: Dynamic covalent chemistry in live cells for organelle targeting and enhanced photodynamic action
Source: Chem Sci. 2022 Feb 4;13(13):3652–60. doi: 10.1039/d1sc04770a (PMC8966643; doi:10.1039/d1sc04770a)
Supplement: SC-013-D1SC04770A-s001 [file SC-013-D1SC04770A-s001.pdf]

Electronic Supplementary Material (ESI) for Chemical Science.
This journal is © The Royal Society of Chemistry 2022

Electronic Supplementary Information

Dynamic covalent chemistry in live cells for organelle targeting and enhanced photodynamic action

Fei Liu,[1] Dmytro I. Danylchuk,[1] Bohdan Andreiuk,[1] Andrey S. Klymchenko[1]*

[1] Laboratoire de Bioimagerie et Pathologies, UMR 7021 CNRS, ITI Chimie des Systèmes Complexes, Université de Strasbourg, 74 route du Rhin, 67401, Illkirch, France

*Corresponding author: andrey.klymchenko@unistra.fr

Experimental Section

Material and Methods

All starting materials for synthesis were purchased from Alfa Aesar or Sigma-Aldrich unless specifically mentioned. MitoTracker Deep Red FM 644/665 were purchased from Thermo Fisher. Nile Red Ketone (NRK),[1] lipid droplets marker SMCy5.5[2] and plasma membrane marker F2N12SM[3] were synthesized as described before. CellTox™ Green assay (Cat.# G8741) was bought in Promega. Milli-Q water (Millipore) was used in all experiments. Synthesis and characterization of all new compounds, including membrane, lipid, and mitochondria targeting agents are described below. NMR spectra were recorded on a Bruker Avance III 400 MHz spectrometer. The following abbreviations are used in the presentation of the NMR data: s, singlet; brs, broad singlet; d, doublet; brd, broad doublet; dt, doublet of triplets; t, triplet; m, multiplet. Mass spectra were obtained using an Agilent Q-TOF 6520 mass spectrometer. Absorption spectra were recorded on Cary 5000 UV-visible spectrophotometer (Agilent). Fluorescence spectra were recorded on an Edinburg FS5 spectrofluorometer. All the spectra were corrected for the wavelength-dependent response function of the detector. Emission measurements were systematically done at rt. The confocal laser scanning microscopy was done by Leica Application Suite. Epi-fluorescence images were obtained from a microscope produced by Nikon Instruments Europe B.V. Cell images were processed using ImageJ 1.52a software (NIH, Bethesda, MD, USA).

In situ reaction in model lipid membranes

Preparation of large unilamellar vesicles (LUVs). A stock solution of dioleoylphosphatidylcholine (DOPC) in chloroform was placed into a round-neck flask, after which the solvent was evaporated in vacuo and phosphate buffer (20 mM, pH 7.4) was added. After all the solid was dissolved a suspension of multilamellar vesicles was extruded by using a Lipex Biomembranes extruder (Vancouver, Canada). The size of the filters was first 200 nm (7 passages) and thereafter 100 nm (10 passages). This generates monodisperse LUVs with a mean hydrodynamic diameter of 120 nm as measured by dynamic light scattering (DLS), using

Malvern Zetasizer Nano ZSP (Malvern, U.K.). The final DOPC concentration in the LUVs was calculated to be 1 mM.

Preparation of nanoemulsions (NEs)

Nanoemulsions were produced by spontaneous nanoemulsification according to protocols described previously.[1] Labrafac oil (Labrafac WL 1349® from Gattefossé, Saint Priest, France) (50 mg) was mixed with the surfactant Kolliphor ELP® (Sigma-Aldrich) (50 mg) in a sonication bath at 40 °C for 10 min. Then, the mixture was homogenized under magnetic stirring at 40 °C for 10 min up to complete homogenization. Finally, NEs were generated with the addition of ultrapure (Milli-Q) water (230 mg). The hydrodynamic diameter of NEs was 90 nm according to DLS.

Model study of *in situ* reactions in LUVs/NEs. 1 mL of LUVs (1 mM) in PBS were mixed with 5 µL of 10 mM stock solution (in DMSO) of membrane targeting compound PM-HZ and incubated for 30 min, then the excess of membrane targeting moiety was removed by dialysis using a dialysis membrane with molecular cut off weight of 14000 Da. Afterwards, 5 µL of 3 mM solution of NRK in DMSO were added and further incubated for 1h at r. t. Then, the mixture was extracted by 500 µL distilled water and 500 µL DCM, DCM phase was collected and concentrated *in vacuo*. The reaction was controlled using TLC by comparing with a control, which was prepared by directly mixing 100 µL of LUVs with 5 µL 3 mM (in DMSO) NRK, followed by the same extraction process. DCM:MeOH=95:5 was used as the eluent. The novel compound, identified by the different polarity in comparing to starting materials, was collected and subjected to mass spectrometry detection. Fluorescence spectra of NRK in LUVs in the presence of the membrane targeting hydrazine PM-HZ were recorded using the following sample preparation: 2 µM NRK in 1 mL in phosphate buffer (pH 7.4) were mixed with 10 µL of the LUVs with PM-HZ, prepared as mentioned above (after the dialysis) and incubated for 30 min. In the two control samples, 2 µM NRK in 1 mL in phosphate buffer were mixed or not with 10 µL of the LUVs (1 mM).

In order to estimate the yield of the hydrazone conjugate of PM-HZ, LD-HZ and Mito-HZ with NRK in LUVs/NEs, the following protocol was used. PM-HZ or Mito-HZ (20 µM) was incubated in 1 mL LUVs (containing 1 mM DOPC) for 20 min at r. t. LD-HZ (20 µM) was incubated in 1 mL NEs (containing 1 mM Labrafac oil) for 20 min at r. t. Then, NRK (10 µM) was added to these solutions and further incubated for 1h at r. t. Finally, the mixture was extracted by 1 mL ethyl acetate twice; the extract was evaporated in vacuum and then purified by TLC (DCM:MeOH=95:5 as an eluent). The formed fractions of NRK-PM-HZ conjugate (with higher polarity, low Rf), NRK-LD-HZ (with lower polarity, high Rf) and NRK-Mito-HZ (with higher polarity, low Rf) and NRK were collected and their absorbance was measured by dissolving in 1 mL MeOH. According to the absorbance of NRK and NRK-PM-HZ, NRK-LD-HZ or NRK-Mito-HZ, the reaction yields of the hydrazone formation were 65 mol%, 63 mol% and 28 mol% for NRK in-situ reaction with PM-HZ, LD-HZ and Mito-HZ, respectively.

Cell experiments

HeLa cells (ATCC® CCL-2) were grown in Dulbecco's modified Eagle's medium (DMEM, Gibco-Invitrogen), supplemented with penicillin (100 U/mL), 1% glycan (100 U/mL) and 10 % FBS at 37 °C in a humidified atmosphere containing 5% CO_2. HeLa cells (ATCC) were then seeded on 36 mm ibidi coverslips at 30,000 cell per well in this DMEM medium with overnight. The DMEM medium was firstly removed and the cells were washed with PBS for three times. Then, medium was changed to Opti-MEM containing or not (in case of control) 100 μM of targeting hydrazides: LD-HZ for lipid droplets, PM-HZ for plasma membrane or Mito-HZ for mitochondria. The cells were incubated at 37 °C in a humidified atmosphere containing 5% CO_2 for 30 min. Then, after washing the cells gently once with PBS, the medium was changed to Opti-MEM containing 1 μM NRK (except 2.5 μM for mitochondria) and further incubated for 30 min at room temperature followed by fluorescence imaging. In the case of protocol without washing out the targeting molecule, the cells were seeded into labtek (8 well chambered coverslips) at 4000 cells per well in this DMEM medium and left overnight. The DMEM medium was then removed and the cells were washed with PBS for three times. The Opti-MEM medium (200 µL) containing certain concentration (1, 5, 20, 100 µM) of HZ-PM was added to HeLa cells. The cells were incubated for 10 min at r.t. followed by addition of NRK (1 µM) and further incubation for 30 min before imaging.

For all co-localization experiments, the cells were seeded into LabTek (8 well chambered coverslips) at 4000 cells per well in the DMEM medium and left overnight. The DMEM medium was then removed and the cells were washed with PBS for three times. Cells were pre-incubated with Opti-MEM medium (200 µL) containing or not (in cases of control) 20 µM corresponding targeting moieties of PM-HZ, LD-HZ or Mito-HZ for 10 min at 37 °C followed by addition of NRK or doxorubicin at 1 µM concentration and further incubated for 30 min. In case of co-localization in plasma membranes, F2N12SM (400 nM) was added after the cells were treated with Mito-HZ and NRK/doxorubicin and incubated for 15 min before imaging. For LDs labeling, LDs marker SMCy5.5 (1 μM) was added to the cells, incubated for 3h at 37 °C before adding targeting hydrazine LD-HZ and NRK/doxorubicin. For mitochondria labeling, MitoTracker Deep Red FM 644/665 (200 nM) was added after the cells were treated with Mito-HZ and NRK/doxorubicin and further incubated for 5 min at 37 °C. Finally, the corresponding medium was changed to Opti-MEM followed by fluorescence imaging. In cases of nucleus staining, Hoechst 33342 (10 µg/mL) in Opti-MEM was used to stain cell nucleus over 1 h and washed with PBS once before all the processes.

For reversibility analysis of reaction of NRK with PM-HZ at plasma membrane interface, the cells in LabTek were pre-incubated with Opti-MEM medium (200 µL) containing or not (in cases of control) 20 µM PM-HZ for 10 min at 37 °C followed by addition of 1 µM NRK and further incubated for 30 min at 37 °C. For experiments with different pH (short incubation time), the cells were washed with PBS three times and the medium was changed to Opti-MEM (pH 7.4) or to phosphate-acetate buffer (pH 5.8). For long incubation in Opti-MEM (pH 7.4), after washing with PBS three times, cell medium was changed to Opti-MEM medium (200 µL) and the cells were incubated for 1 h at 37 °C. For each experimental condition, just before imaging the cells were incubated for 5 min at 37 °C with reference plasma membrane probe F2N12SM (400 nM).[3]

Fluorescence imaging of NRK was done with the excitation wavelength at 488 nm, and fluorescence emission was collected at two different spectral channels: 550-600 nm and 600-670 nm. In the colocalization measurements with LDs marker SMCy5.5, for NRK channel, excitation wavelength was set at 488 nm and emission wavelength was collected in the range of 550-600 nm. For doxorubicin channel, the excitation wavelength was at 488 nm and fluorescence emission was collected in range of 520-650 nm (images of for membrane and mitochondria targeting were collected over average of 20 frames for this channel due to low brightness of doxorubicin). For the SMCy5.5 and MitoTracker Deep Red FM 644/665 channel, the excitation wavelength was set at 635 nm and the emission wavelength was collected in the range of 650-800 nm.
Colocalization analysis was conducted in two ways. The first way was by calculating Pearson's and Mander's correlation coefficients. This was conducted by using an ImageJ plugin named "Just Another Colocalisation Plugin (JACoP)". A certain threshold for both Green and Red channels was applied to select the ROI area for the calculation of Pearson's and Mander's correlation coefficients. The second way was to calculate the ratio of the mean fluorescence intensity from organelle areas versus the whole cells. The organelle areas were selected by using fluorescence image of corresponding reference markers, F2N12SM for plasma membrane, SMCy5.5 for lipid droplet or Mito Tracker Deep Red FM for mitochondria. Cell selection and calculation of the mean fluorescence intensity were done using ImageJ.

Phototoxicity study

HeLa cells (ATCC® CCL-2) were grown in Dulbecco's modified Eagle's medium (DMEM, Gibco-Invitrogen), supplemented with penicillin (100 U/mL), 1% glycan (100 U/mL) and 10 % FBS at 37 °C in a humidified atmosphere containing 5% CO_2. HeLa cells (ATCC) were then seeded on 36 mm coverslips at 30,000 cell per well in this DMEM medium with overnight. The DMEM medium were first removed and washed with PBS for three times before changing the medium to Opti-MEM (as control) or Opti-MEM containing PM-Hz, Mito-HZ or LD-HZ (100 μM), then the cells were incubated at 37 °C in a humidified atmosphere containing 5% CO_2 for 30 min. After that the cell were washed with PBS and the medium was changed to Opti-MEM containing 1 μM of NRK, followed by an incubation for 30 min at r. t. To understand PDT effect when NRK is targeted to different cell organelles, we used CellTox™ Green, a nucleus maker for monitoring cell death induced by membrane disruption. After targeting NRK to cell organelles with corresponding ligands, the dishes with cells were washed once with PBS and subjected to illumination with excitation of 550 nm (50% LED source power) continuously over 1 min followed by changing the medium to 1 mL CellTox™ Green assay containing 12 µg/mL Hoechst nucleus staining agent and incubated for 30 min. The transmission and fluorescence (CellTox™ Green channel) images were taken before and after illumination using 20x air objective Nikon CFI Plan Apo, NA = 0.75 (for data in Figure 3) or 10x air objective Nikon Plan Fluor NA = 0.3 (for data in Figure S5); excitation: 470 nm, emission filter: 531/40 nm for CellTox™ Green channel and excitation: 405 nm, emission filter: 475/50 nm for Hoechst 33342 channel. To observe the cell morphology change induced by phototoxicity, cell images (both bright field and epi-fluorescence channel (4% power, 300 ms

integration period) with excitation of 550 nm and filter of 600/50 nm) were firstly taken before the application of high source power. Then video was prepared by taking images of the bright field transmission channel and epi-fluorescence channel (source power: 30% for lipid droplets, plasma membrane and their controls; 50 % for mitochondria and its control) and short integration period (30 ms) continuously for 30 slices over 10 min. After the video recording, images were taken in dic channel (300 ms) and epi channel (4% power, 300 ms integration period). This cellular imaging studies were done using epi-fluorescence mode with a Nikon Ti-E inverted microscope, equipped with CFI Plan Apo ×60 oil (numerical aperture = 1.4) objective, and a Hamamatsu Orca Flash 4 sCMOS camera.

Synthesis of targeting hydrazides

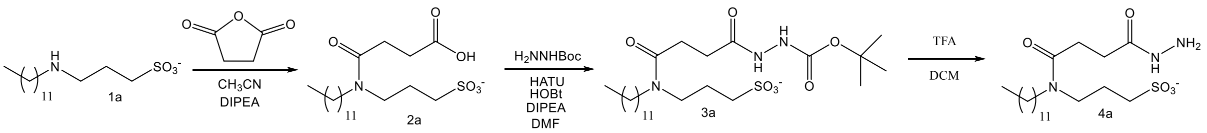

Scheme S1. Synthesis route for PM-HZ (**4a**).

Compound **1a** was prepared as described previously.[4]

Compound 2a. 400 mg of compound **1a** were dissolved in 7 mL of dry CH_3CN together with 589 mg (3.5 equiv., 794 µL) of DIPEA. After that, a solution of 196 mg (1.5 eq.) of succinic anhydride 3 mL of dry CH_3CN was added dropwise and the resulting mixture was stirred for 12h (control by TLC) under Ar atmosphere. After the reaction the solvent was evaporated *in vacuo* and the crude product was purified by column chromatography (SiO_2, DCM:MeOH:HCOOH 80:20:2).Yield: 550 mg as a colourless solid (DIPEA salt is present, its signals are not presented). ^{1}H NMR (400 MHz, MeOH-D4) δ ppm 3.49 (2H, dt), 3.36 (2H, dt), 2.86 (2H, dt), 2.68 (2H, m), 2.58 (2H, t), 2.06 (2H, m), 1.60 (2H, brd), 1.30 (18 H, brs), 0.81 (3H, t). Splitting of d, f, g was due to presence of amide rotamers, which disappeared under high temperature. ^{13}C NMR (400 MHz, MeOH-D4) δ ppm 176.70, 173.94, 166.88, 55.91, 54.96, 50.30, 49.15, 48.04, 47.42, 46.26, 43.89, 33.19, 30.87, 30.71, 30.61, 30.59, 30.50, 30.00, 29.16, 28.88, 28.21, 28.06, 25.58, 24.66, 23.86, 18.11, 17.85, 14.58, 13.26. Chemical Formula: [$C_{19}H_{36}N_3O_6S^-$] m/z (M) calc. 406.2263 MS for m/z [M^-+2H^+]$^+$ found 408.2432.

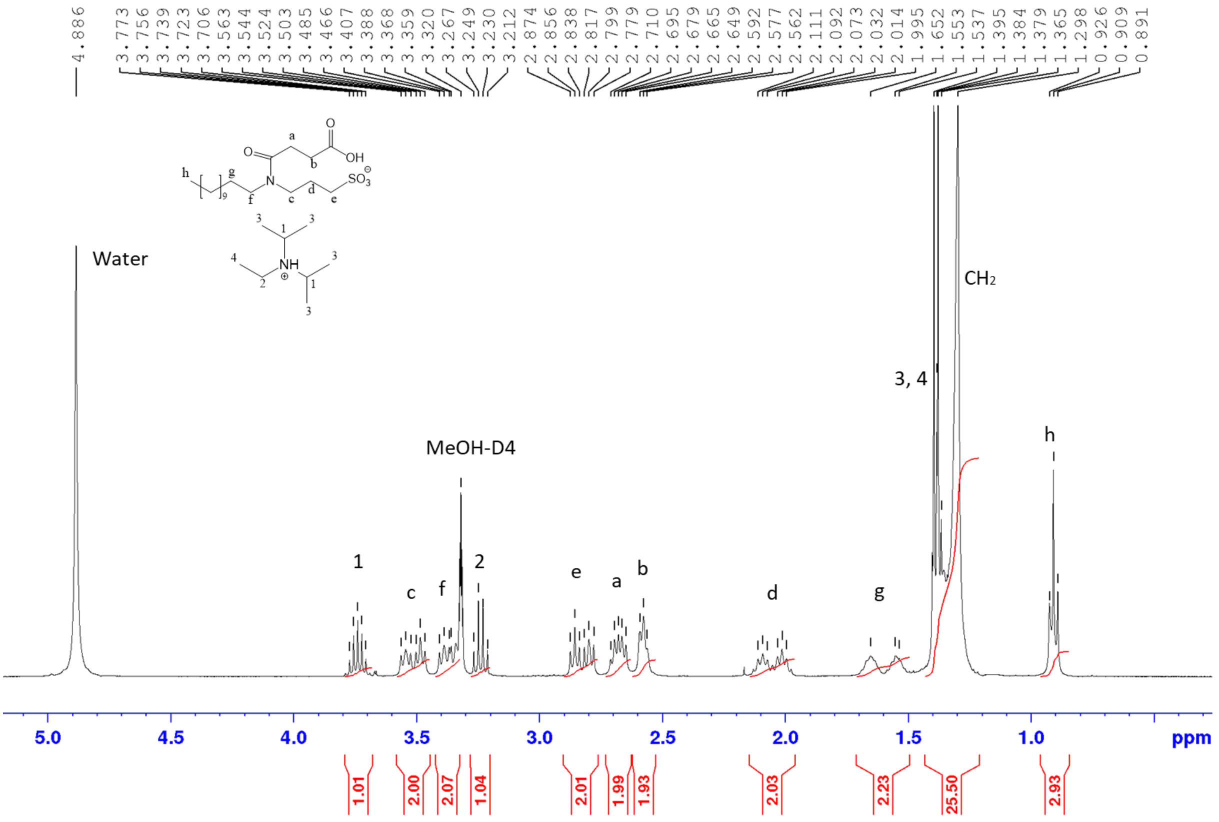

^{1}H-NMR spectrum of **2a** in MeOH-D4.

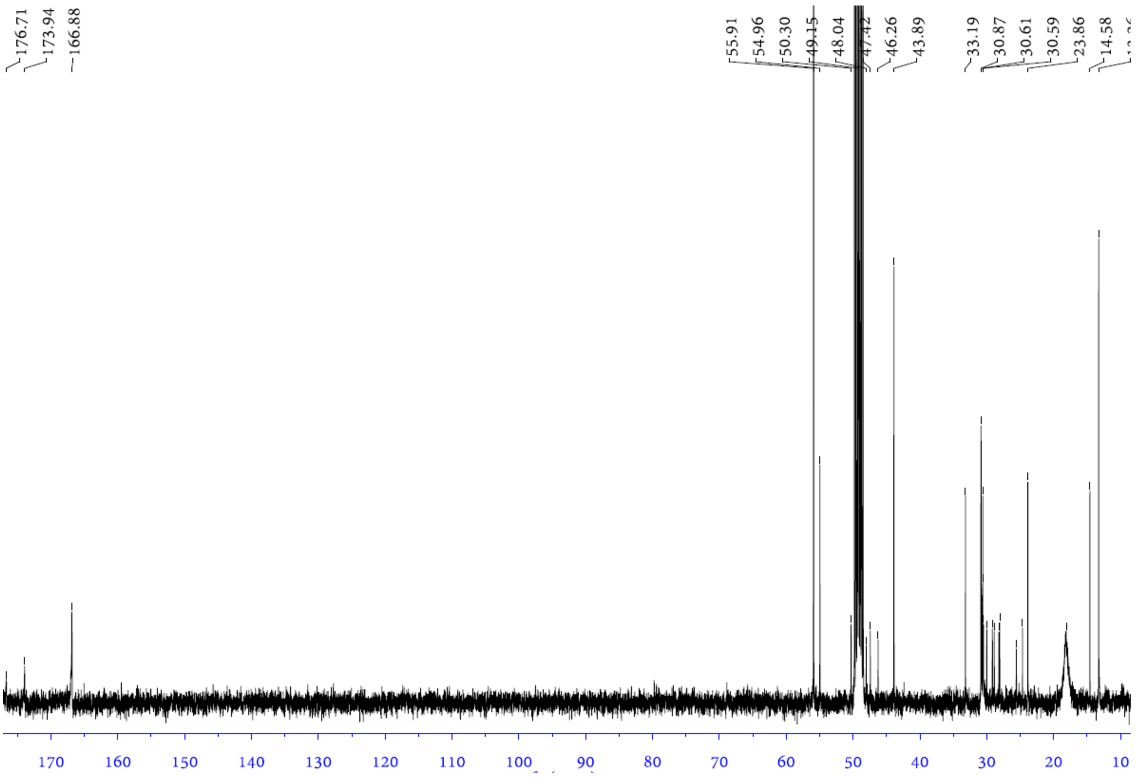

^{13}C-NMR spectrum of **2a** in MeOH-D4.

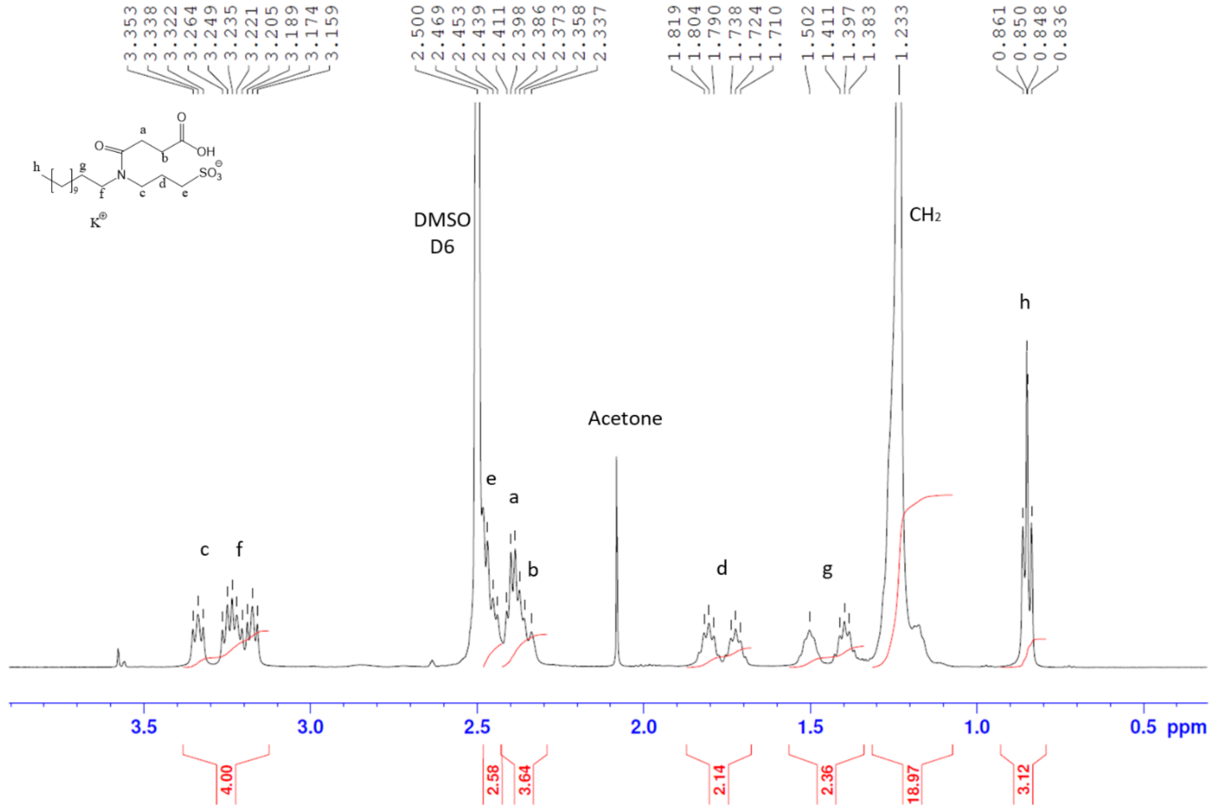

^{1}H-NMR spectrum of **2a** in DMSO (D6) under room temperature after ion exchange of protonated DIPEA with K^+ ion.

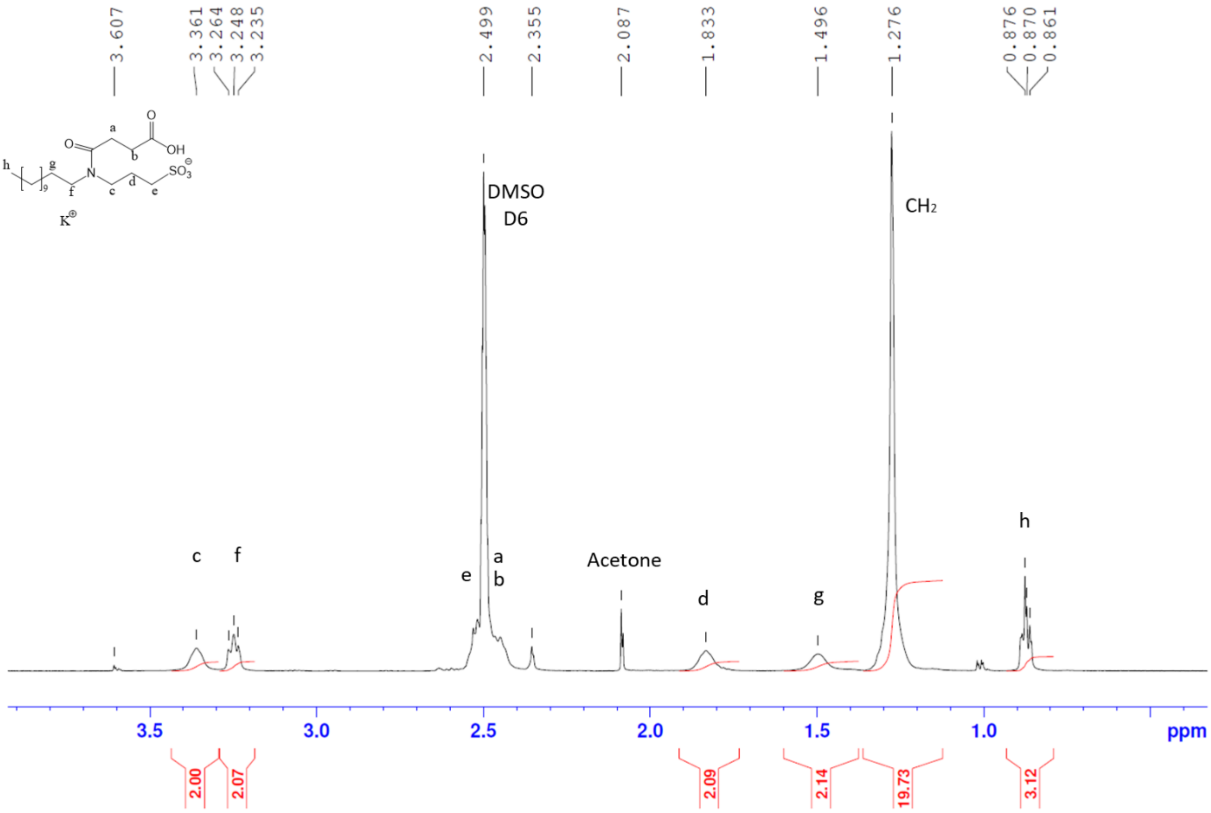

^{1}H-NMR spectrum of **2a** under high temperature (375 K) after ion exchange of protonated DIPEA with K^+ ion in DMSO (6d).

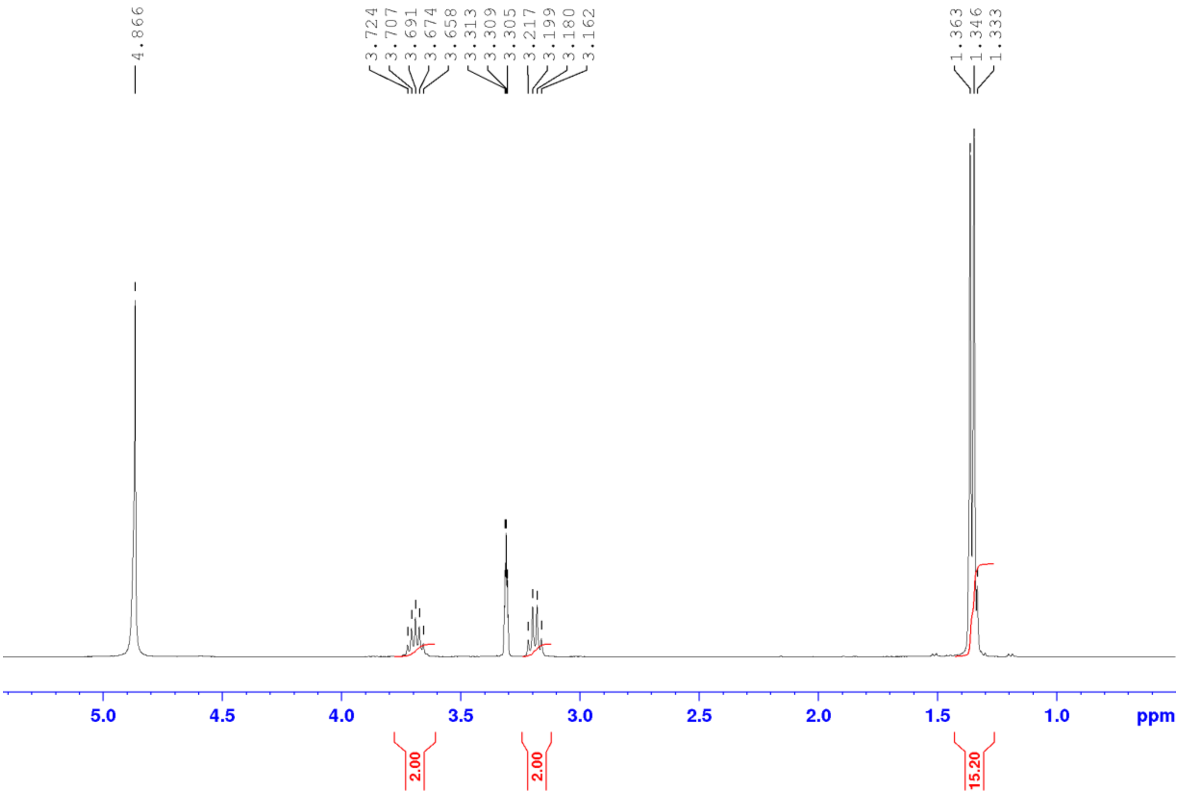

^{1}H-NMR spectrum of protonated N,N-diisopropylethylamine (DIPEA) in TFA in MeOH-D4.

Compound 3a. 200 mg of compound **2a** were dissolved in 3 mL of dry DMF together with 238 mg (1.5 eq.) of HATU, 28 mg (0.5 eq.) of HOBt and 215 mg (4 eq., 291 µL) of DIPEA under Ar atmosphere. After 5 minutes, a solution of 83 mg (1.5 eq.) of tert-Butyl carbazate in 1.5 mL of dry DMF was added and the mixture was stirred for 24h (control by TLC) under Ar atmosphere. After the reaction the solvent was evaporated *in vacuo* and the crude product was purified by column chromatography (SiO_2, DCM:MeOH 80:20, redone twice).Yield: 165 mg as a colourless solid (DIPEA salt is present, its signals are not presented). ^{1}H NMR (400 MHz, MeOH-D4) δ ppm 3.50 (2H, dt), 3.37 (2H, dt), 2.82 (2H, dt), 2.74 (2H, m), 2.53 (2H, t), 2.05 (2H, m), 1.60 (2H, brd), 1.47 (9H, m), 1.30 (18H, brs), 0.91 (3H, t). ^{13}C NMR (400 MHz, MeOH-D4) δ ppm 174.98, 174.92, 173.85, 173.72, 158.11, 157.95, 82.18, 81.96, 56.01, 50.01, 49.15, 48.06, 47.46, 46.30, 43.96, 33.19, 30.87, 30.71, 30.64, 30.59, 30.26, 30.04, 29.39, 29.23, 28.87, 28.71, 28.25, 28.09, 25.62, 24.50, 23.85, 18.89, 17.45, 14.57. HRMS for Chemical Formula: [$C_{24}H_{46}N_3O_7S^-$] m/z (M) calc. 520.3056, found 520.3051.

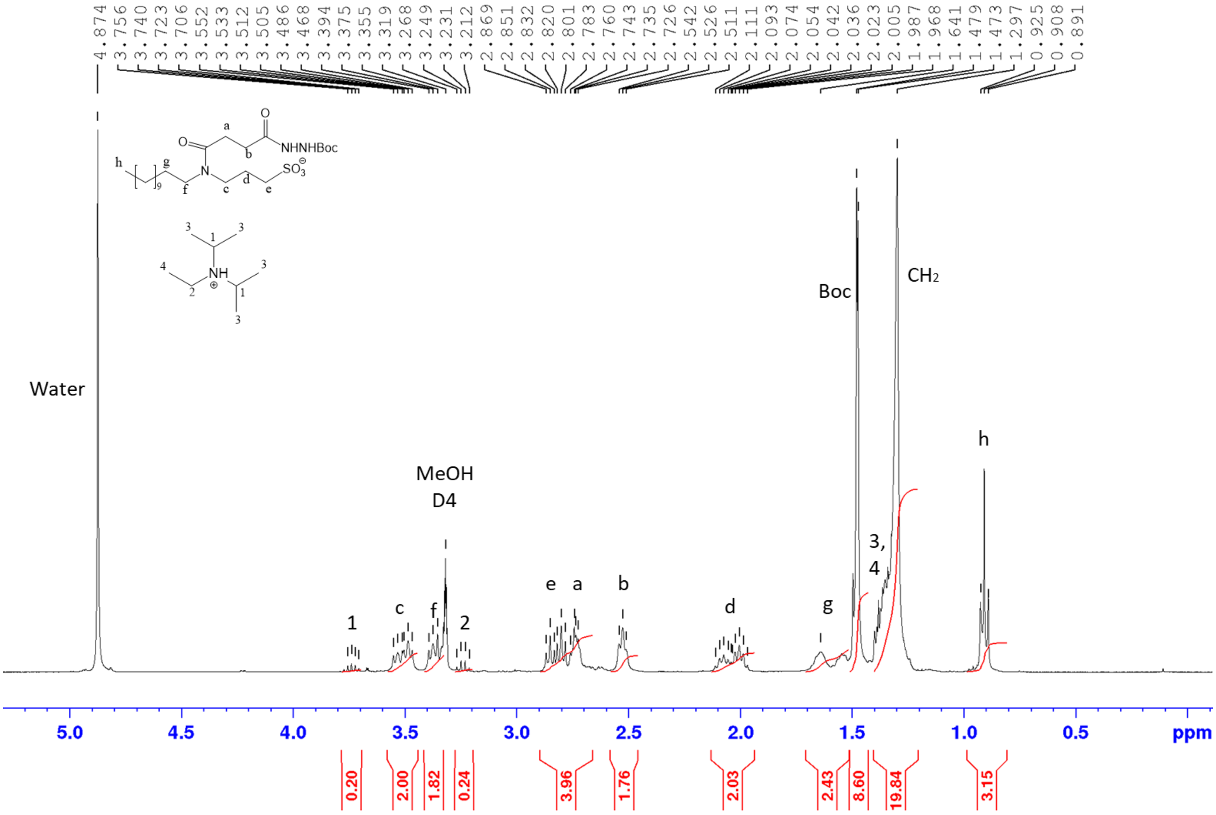

^{1}H-NMR spectrum of **3a** in MeOH-D4.

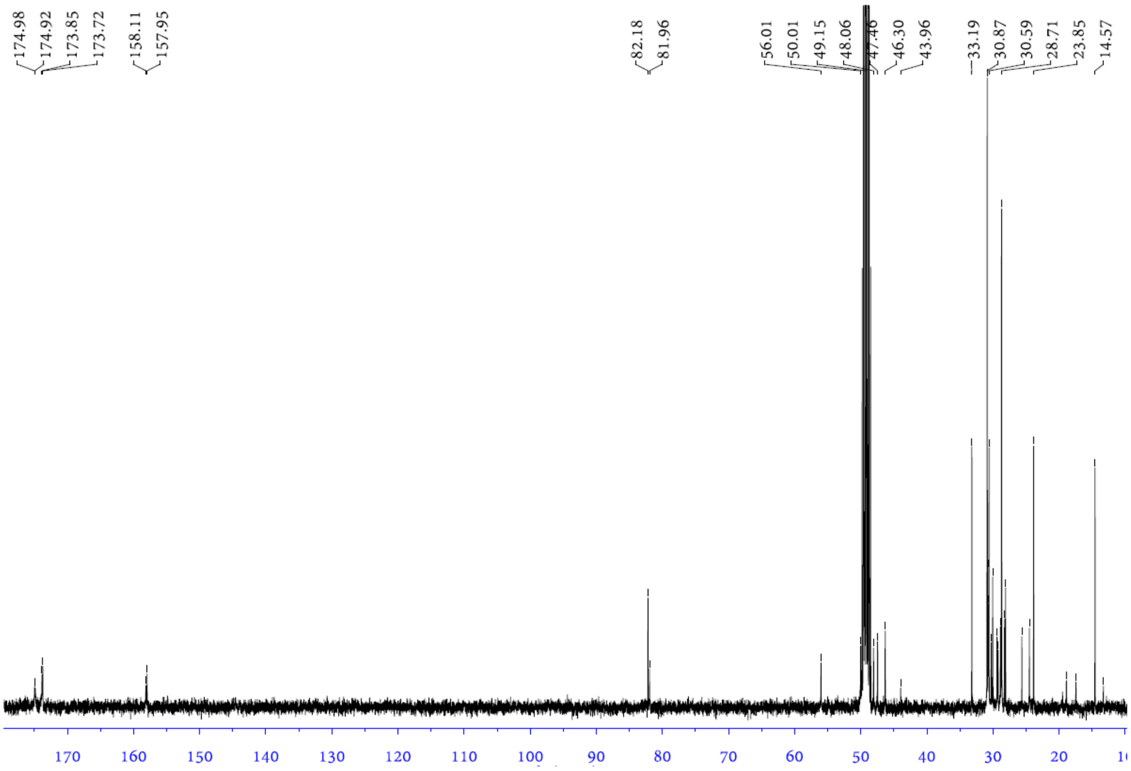

^{13}C-NMR spectrum of **3a** in MeOH-D4.

Compound 4a (PM-HZ). To the solution of **3a** (165 mg, 0.31 mmol) in DCM (5 mL), TFA (5 mL) and 1 drop of MiliQ water were added and stirred at room temperature for 2 h. After confirming the reaction completely proceeded with TLC, the solvents were evaporated under reduced pressure, and then the residue was dissolved in MeOH and evaporated 3 times to remove TFA and give desired product **4a** as a colorless solid (153 mg) (DIPEA salt is present, its signals are not presented). ^{1}H NMR (400 MHz, MeOH-D4) δ ppm 3.51 (2H, brd), 3.39 (2H, brs), 2.80 (4H, m), 2.59 (2H, brs), 2.04 (2H, brd), 1.59 (2H, brd), 1.30 (18H, brs), 0.91 (3H, t). ^{13}C NMR (400 MHz, MeOH-D4) δ ppm 173.94, 173.91, 166.88, 55.91, 54.96, 50.30, 49.15, 48.04, 47.42, 46.26, 43.89, 33.19, 30.87, 30.71, 30.61, 30.59, 30.50, 30.00, 29.16, 28.88, 28.21, 28.06, 25.58, 24.66, 23.86, 18.11, 17.85, 17.67, 14.58, 13.26. HRMS for Chemical Formula: [$C_{19}H_{38}N_3O_5S^-$] m/z (M) calc. 420.2532, found 420.2534.

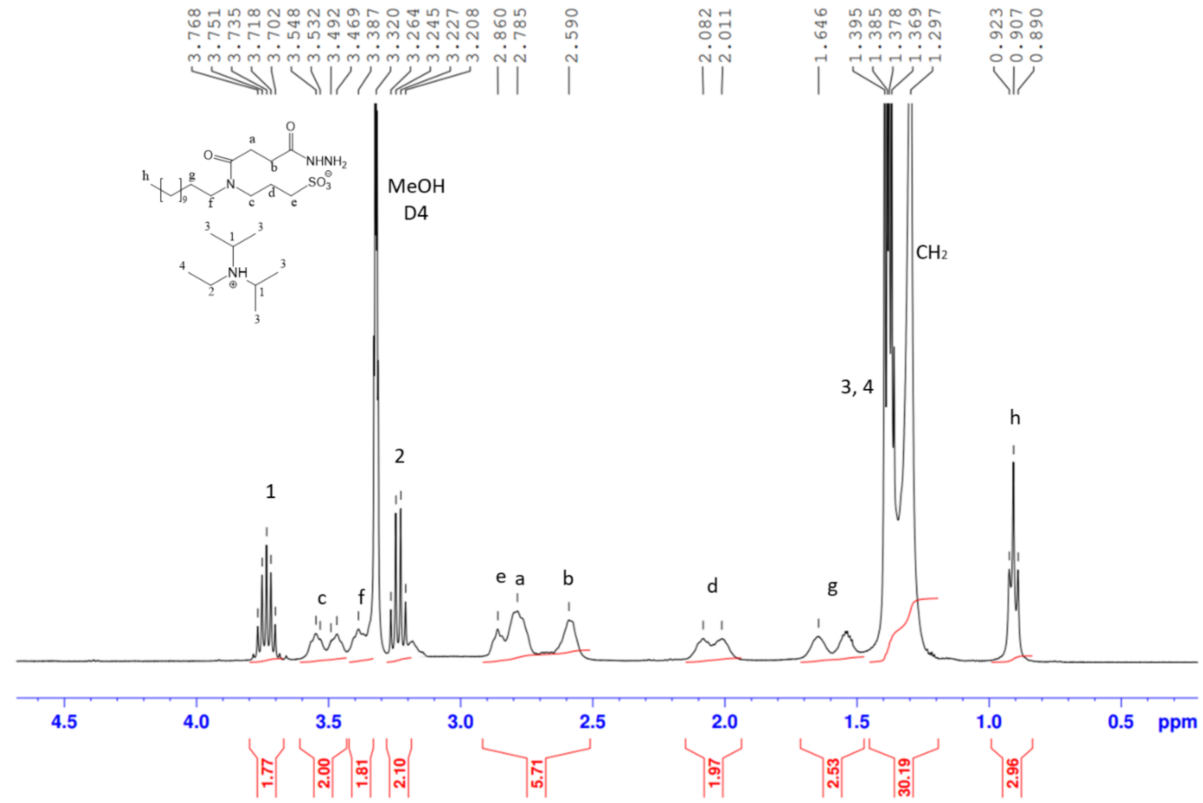

^{1}H-NMR spectrum of **4a** in MeOH-D4.

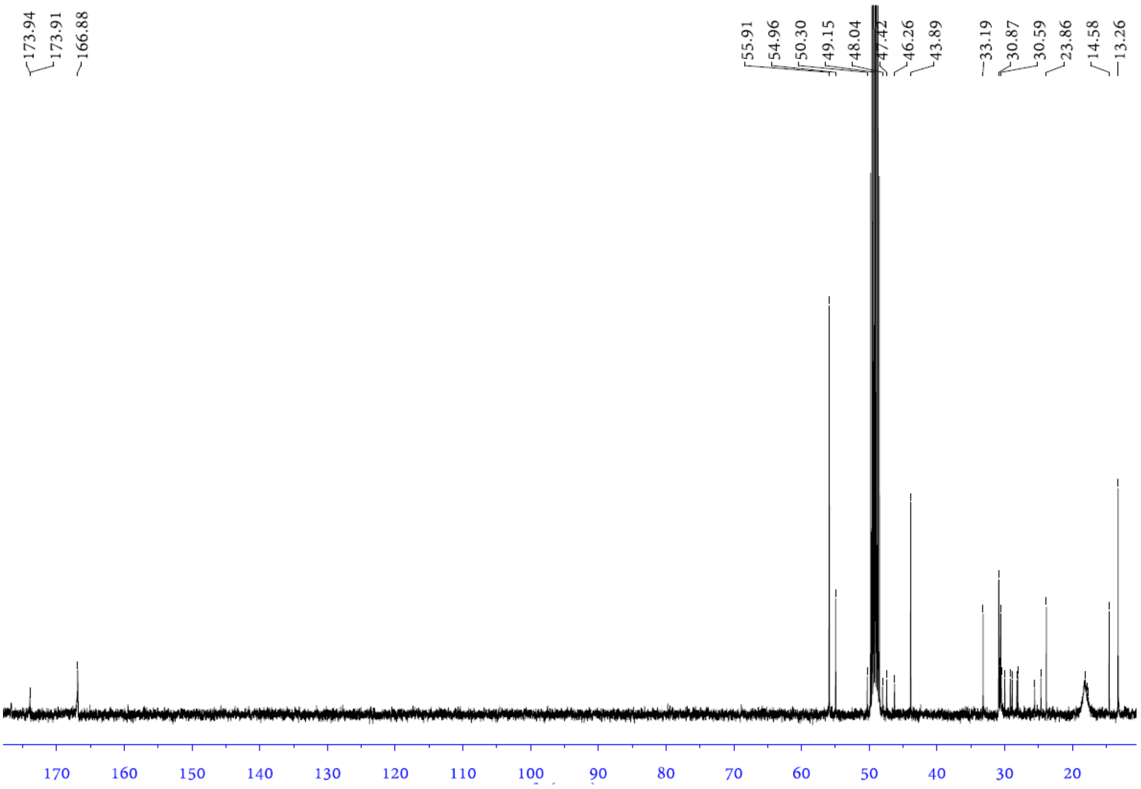

^{13}C-NMR spectrum of **4a** in MeOH-D4.

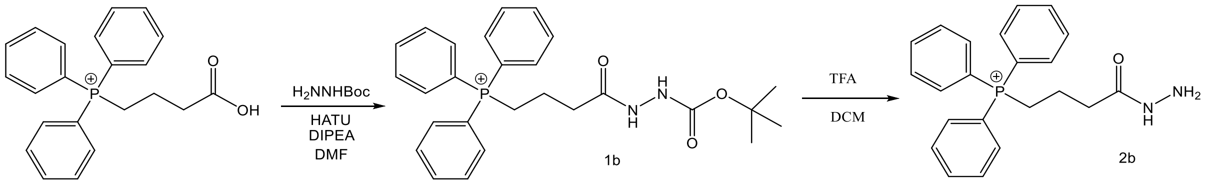

Scheme S2. Synthesis route for Mito-HZ (**2b**).

Compound 1b. 500 mg of (3-carboxypropyl)triphenylphosphonium bromide were dissolved in 8 mL of dry DMF together with 465 mg (1.05 eq.) of HATU and 450 mg (3 eq., 610 µL) of DIPEA. After 5 minutes, a solution of 161 mg (1.05 eq.) of tert-Butyl carbazate in 2 mL of dry DMF was added and the mixture was stirred for 24h (control by TLC) under Ar atmosphere. After the reaction the solvent was evaporated *in vacuo*, then the solid residue was dissolved in MeOH and precipitated with Et_2O, filtered and washed with Et_2O (redone twice). After that, the solid residue was dissolved in DCM, washed with water (x2) and brine, dried over Na_2SO_4 and the solvent was evaporated *in vacuo*. The crude product was purified by gradient column chromatography (SiO_2, DCM:MeOH 90:10 to 80:20).Yield: 70 mg as a colourless solid. ^1H NMR (400 MHz, MeOH-D4) δ ppm 7.81 (15H, m), 3.54 (2H, t), 2.48 (2H, t), 1.99 (2H, m), 1.47 (9H, s). ^{13}C NMR (400 MHz, MeOH-D4) δ ppm 174.30, 158.09, 136.48, 136.46, 135.01, 134.93, 131.74, 131.64, 120.23, 119.54, 82.16, 49.15, 34.38, 34.23, 28.68, 22.27, 21.84, 19.98. HRMS for Chemical Formula: [$C_{27}H_{32}N_2O_3P^+$] m/z (M) calc. 463.2151, found 463.2657.

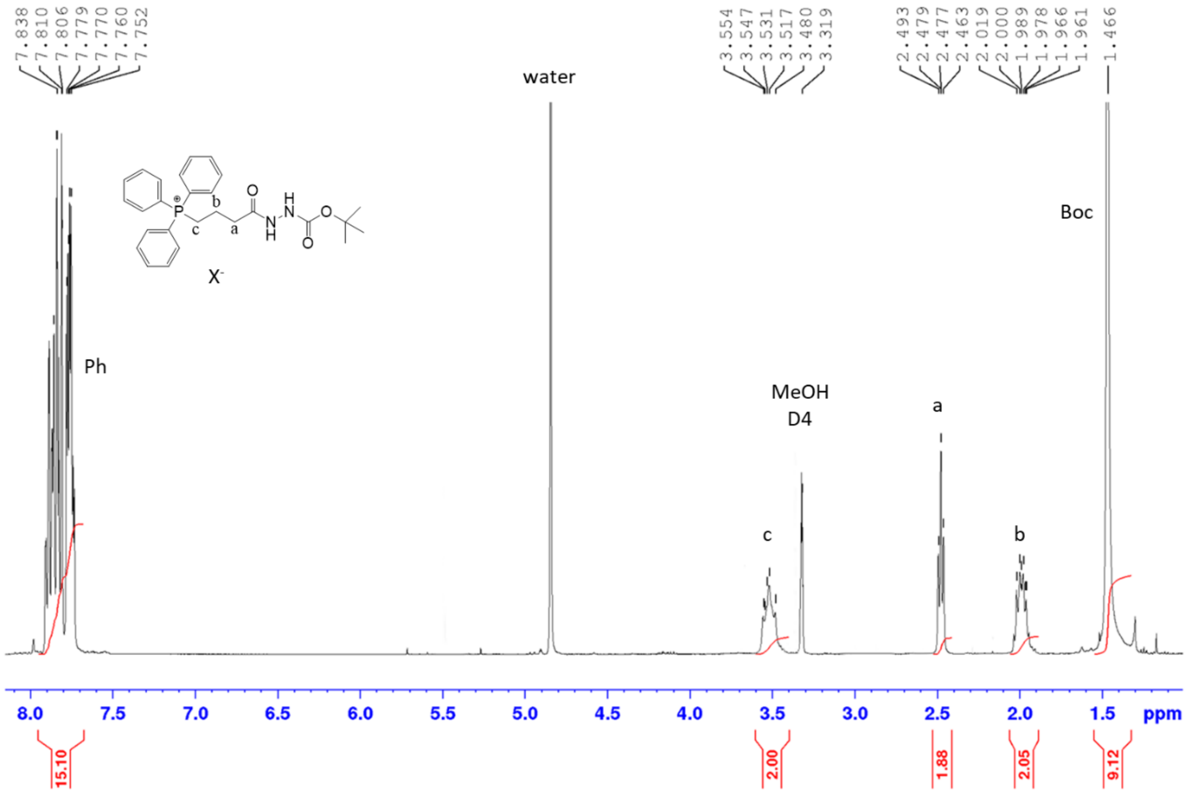

^1H-NMR spectrum of **1b** in MeOH-D4.

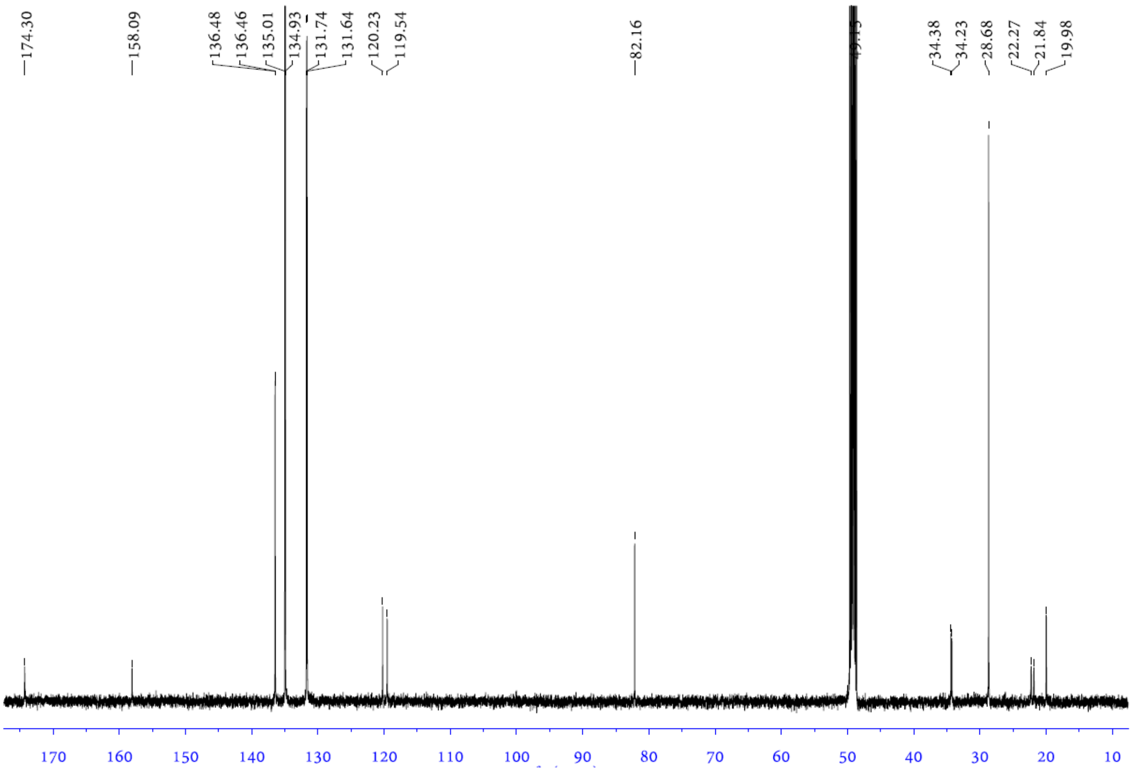

^{13}C-NMR spectrum of **1b** in MeOH-D4.

Compound 2b (Mito-HZ). To the solution of **1b** (70 mg, 0.15 mmol) in DCM (2 mL), TFA (2 mL) and 1 drop of MiliQ water were added and stirred at room temperature for 2 h. After confirming the reaction completely proceeded with TLC, the solvents were evaporated under reduced pressure, and then the residue was dissolved in MeOH and evaporated 3 times to remove TFA and give desired product **4a** as a colorless solid (54 mg). ^{1}H NMR (400 MHz, MeOH-D4) δ ppm 7.83 (15H, m), 3.47 (2H, m), 2.61 (2H, t), 2.01 (2H, pentet). ^{13}C NMR (400 MHz, MeOH-D4) δ ppm 136.58, 136.55, 135.01, 134.91, 131.81, 131.68, 131.49, 120.17, 119.30, 117.60, 114.77, 49.15, 22.67, 22.15, 19.25. HRMS for Chemical Formula: [$C_{22}H_{24}N_2OP^+$] m/z (M) calc. 363.1626, found 363.2060. This compound was further characterized by RP-HPLC by using C4 column (diameter 4.6 mm, length 250 mm, 5 µM pore size) at flow rate of 1 mL/min, 45 °C, 50% /50% of water/acetonitrile on an Agilent 1290 Infinity II LC System.

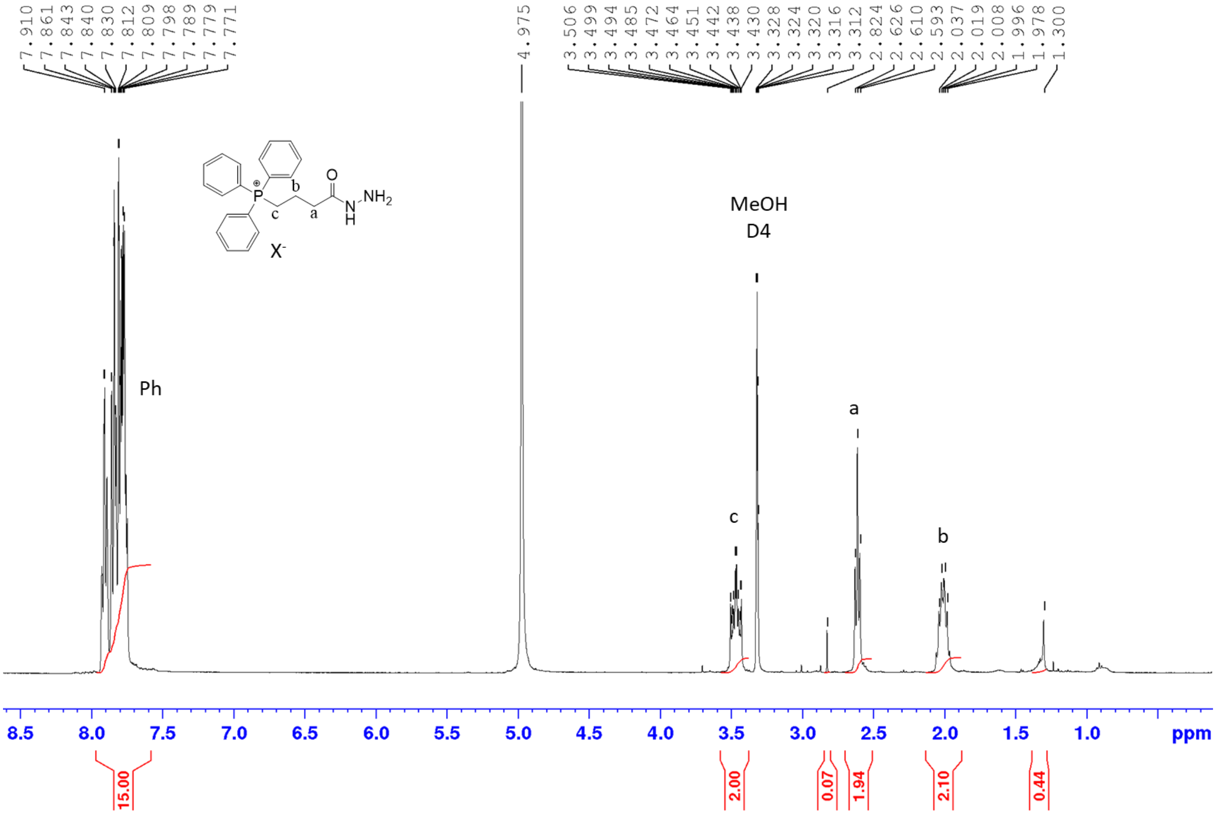

^{1}H-NMR spectrum of **2b** MeOH-D4.

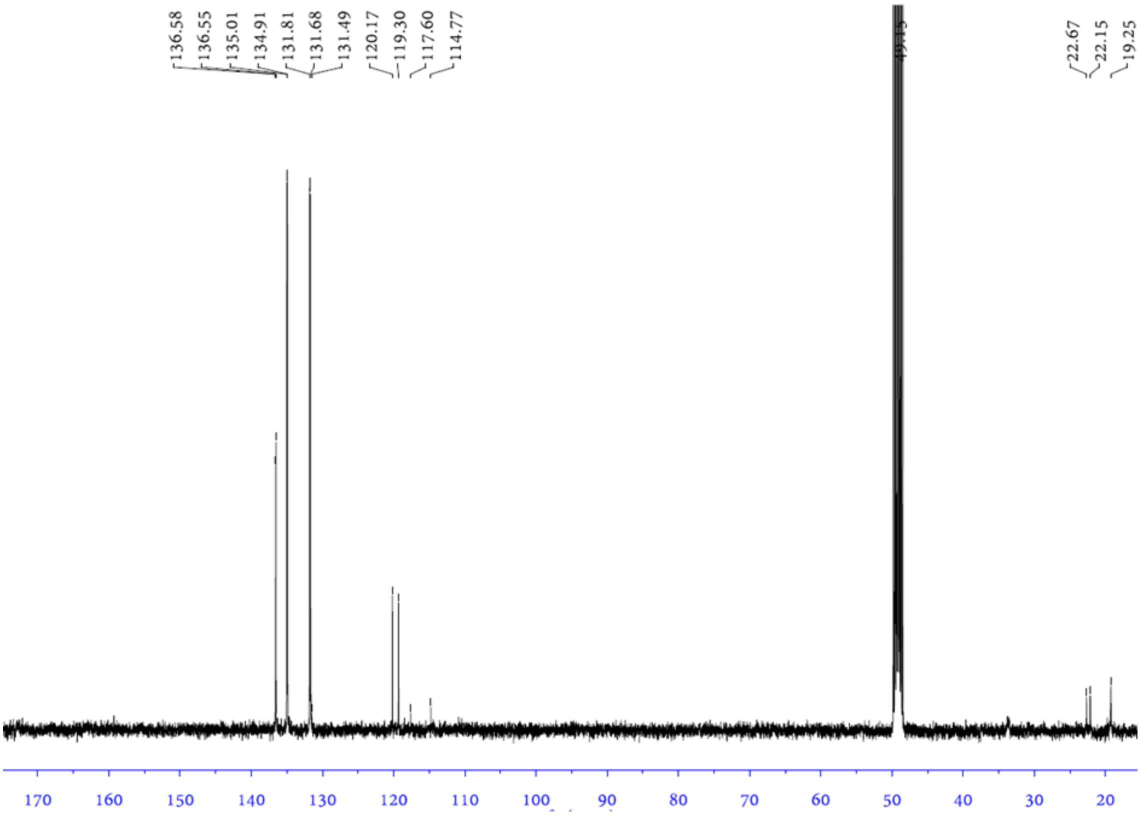

^{13}C-NMR spectrum of **2b** MeOH-D4.

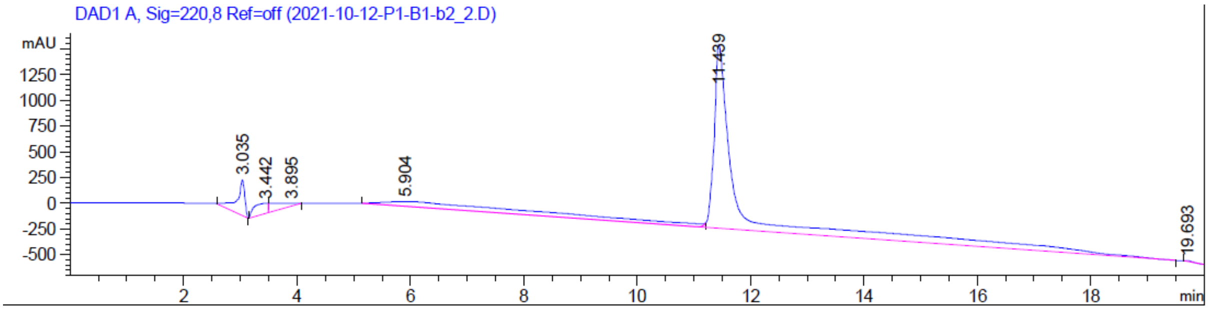

RP-HPLC profile of **2b**.

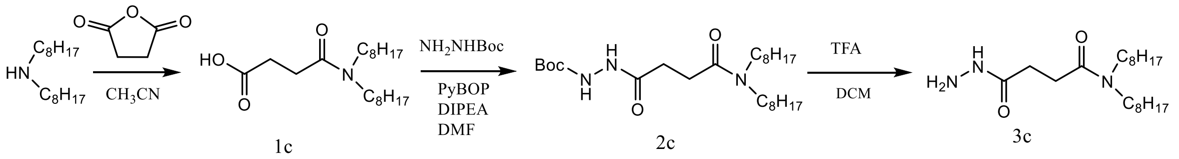

Scheme S3. Synthesis route for LD-HZ (**3c**).

Compound 1c. To the solution of succinic anhydride (192 mg, 1.92 mmol) in THF (20 mL) was added 1,1-Dioctylamine (250 mg, 1.04 mmol), then mixture was refluxed overnight under argon atmosphere. The solvent was evaporated in vacuo, then resulting residue was purified by recrystallization from dioxane to give desired product as a colorless solid (243 mg, 68 %). ^{1}H NMR (400 MHz, $CDCl_3$) δ ppm 3.23 (2H, t), 3.18 (2H, t), 2.62 (2H, m), 2.59 (2H, m), 1.51 (2H, m), 1.45 (2H, m), 1.21 (20H, m), 0.81 (6H, m). ^{13}C NMR (400 MHz, $CDCl_3$) δ ppm 176.48, 171.97, 77.23, 53.61, 48.36, 47.72, 46.67, 31.97, 31.92, 31.90, 30.48, 29.53, 29.46, 29.41, 29.36, 29.30, 29.24, 29.02, 28.33, 27.86, 27.21, 27.08, 26.94, 25.97, 22.80, 22.78, 14.24, 14.22. HRMS for Chemical Formula [$C_{20}H_{39}NO_3^-$] m/z (M) calc. 341.2929, found m/z for [$M^- + 2Na^+ - H^+]^+$ 386.3101.

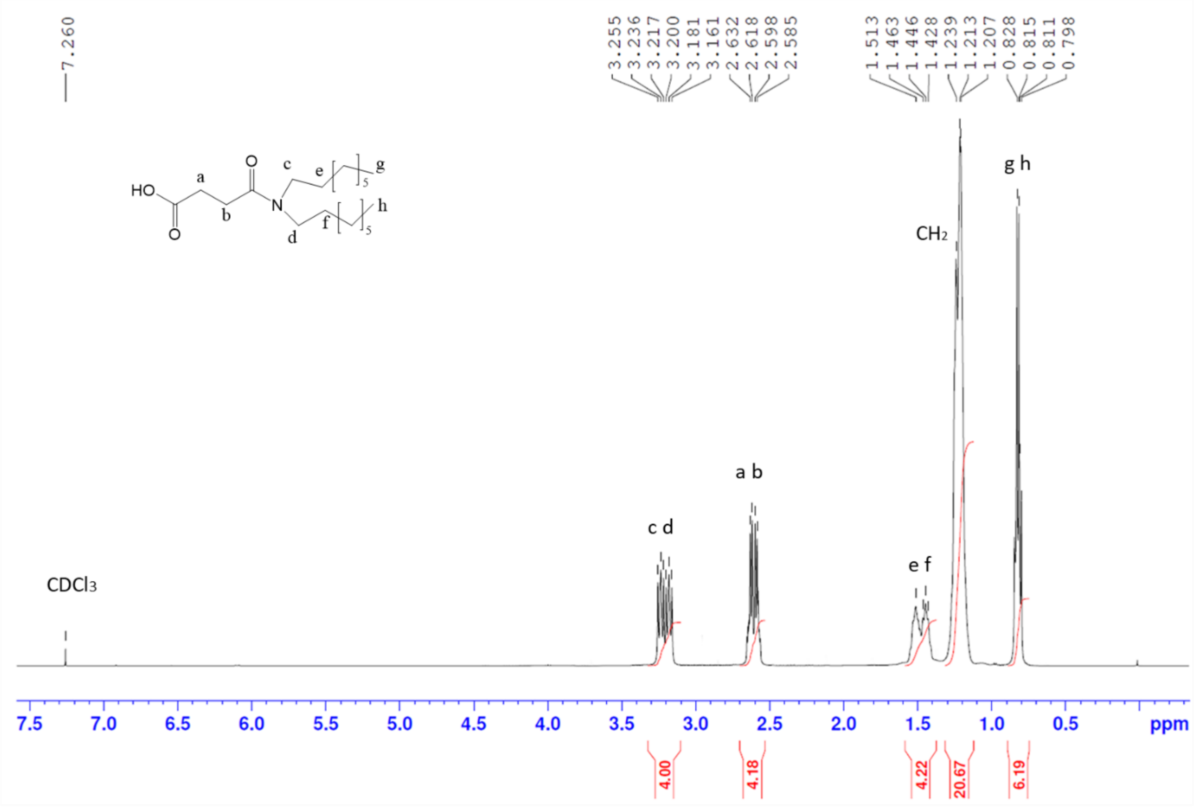

^{1}H-NMR spectrum of **1c** in $CDCl_3$.

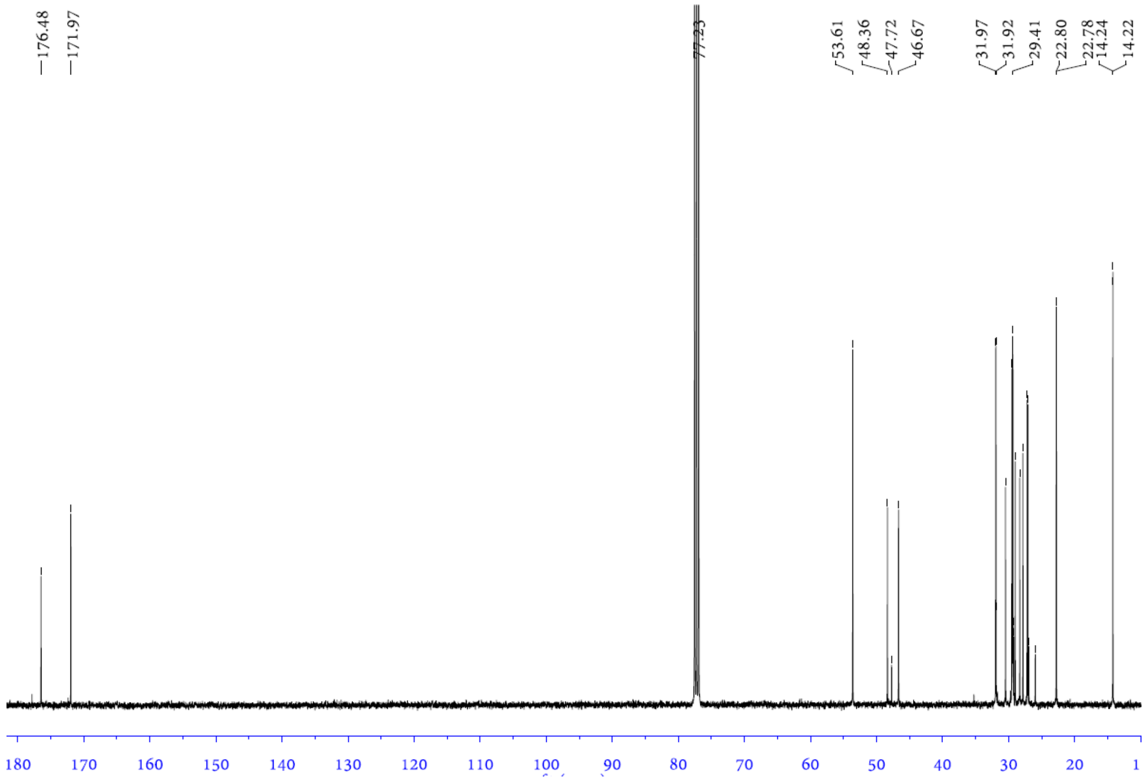

^{13}C-NMR spectrum of **1c** in $CDCl_3$.

Compound 2c. To the solution of **1c** (243 mg, 0.71 mmol) in DMF (10 mL), tert-butylcarbazate (159 mg 1.21 mmol), PyBOP (630 mg, 1.21 mmol), and DIPEA (1.11 mL, 6.4 mmol (9 eq)) were added and stirred at room temperature overnight under argon atmosphere. The solvent was evaporated in vacuo, then resulting residue was purified by flash column chromatography on silica gel (eluent EtOAc/Hepane 50:50) to give desired product as a colorless solid (163 mg, yield 50%). ^{1}H NMR (400 MHz, $CDCl_3$) δ ppm 3.17 (4H, m), 2.58 (2H, m), 2.51 (2H, m), 1.3-1.5 (13H, m), 1.20 (20H, m), 0.79 (6H, m). ^{13}C NMR (400 MHz,$CDCl_3$) δ ppm 172.38, 171.01, 155.21, 81.17, 77.00, 60.33, 56.88, 53.43, 49.97, 47.84, 46.52, 46.17, 31.68, 31.63, 29.26, 29.17, 29.12, 29.07, 28.67, 28.23, 28.10, 28.04, 27.91, 27.60, 26.94, 26.87, 26.79, 26.73, 22.50, 22.48, 13.86. HRMS for Chemical Formula $[C_{20}H_{39}NO_3]^-$ m/z (M) calc 455.3723, found m/z for $[M+Na^+]^+$ 478.4062.

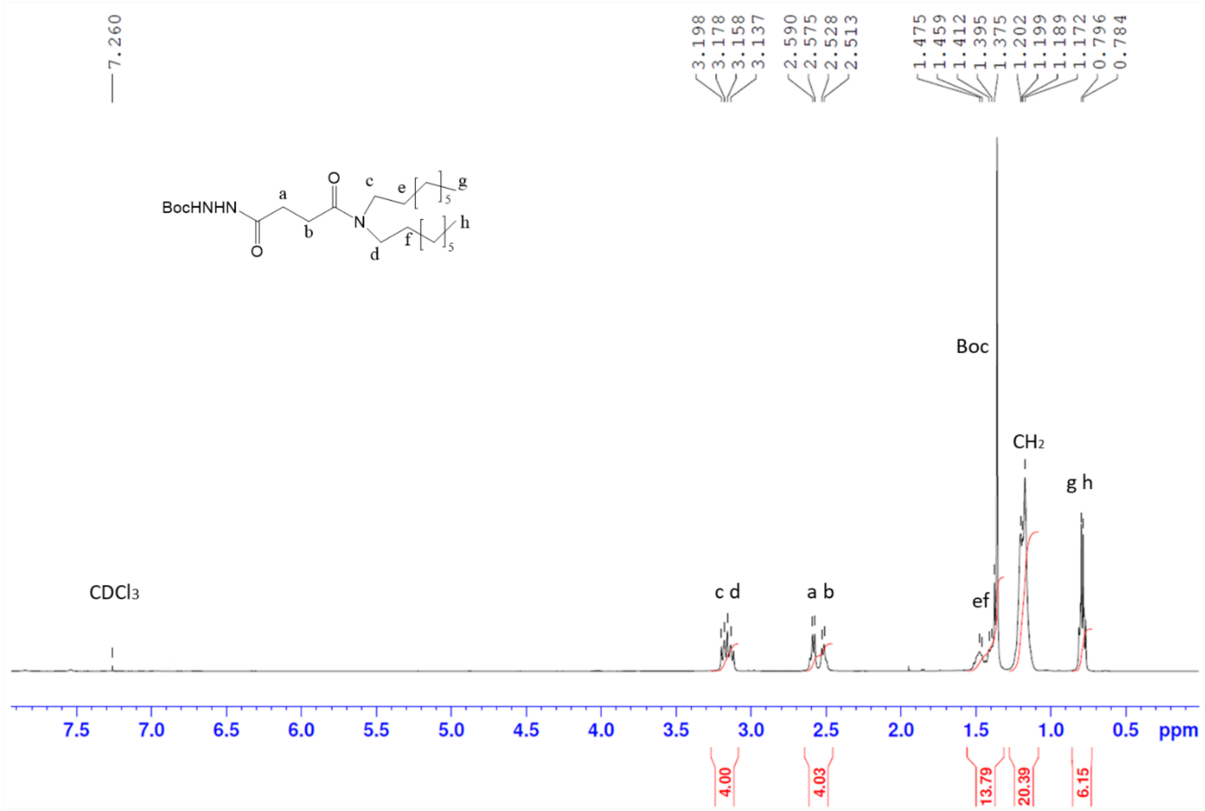

^{1}H-NMR spectrum of **2c** in $CDCl_3$.

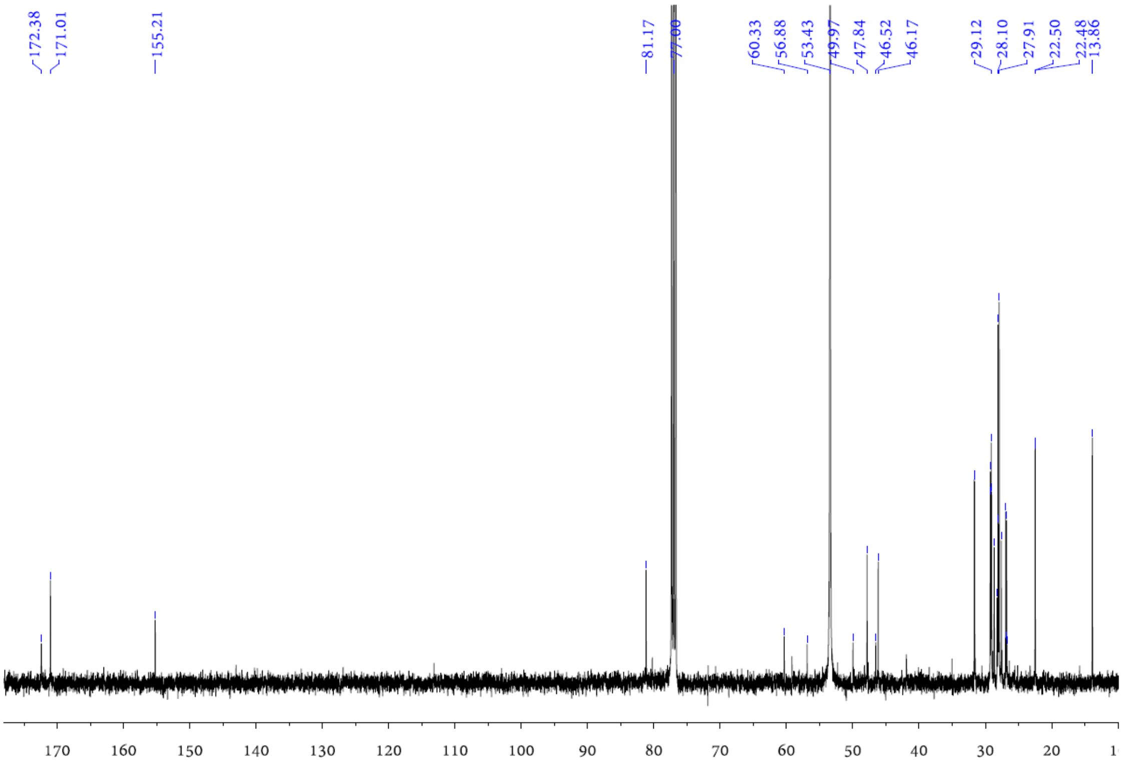

^{13}C-NMR spectrum of **2c** in $CDCl_3$.

Compound 3c (LD-HZ). To the solution of **2c** (163 mg, 0.48 mmol) in DCM (10 mL), TFA (10 mL) and 2 drops of MiliQ water were added and stirred at room temperature for 2 h. After confirming the reaction completely proceeded with TLC, the solvents were evaporated under reduced pressure, and then the residue was dissolved in MeOH and evaporated 3 times to remove TFA and give desired product **3c** as a colorless solid (158 mg). ^{1}H NMR (400 MHz, $CDCl_3$) δ ppm 3.22 (4H, m), 2.68 (2H, brs), 2.56 (2H, brs), 1.50 (4H, brd), 1.26 (20H, m), 0.87 (6H, t). Splitting of protons of c, d and e, f were due to presence of amide rotamers; the splitting disappeared under high temperature. ^{13}C NMR (400 MHz, $CDCl_3$) 172.99, 171.69, 77.48, 77.16, 76.84, 48.56, 46.90, 31.91, 31.86, 29.42, 29.33, 29.30, 28.69, 27.64, 27.10, 27.01, 22.72, 14.16, 14.15. 3HRMS for Chemical Formula: [$C_{20}H_{41}N_3O_2$] m/z calc. 355.3199, found m/z (M+Na$^+$) 378.3163.

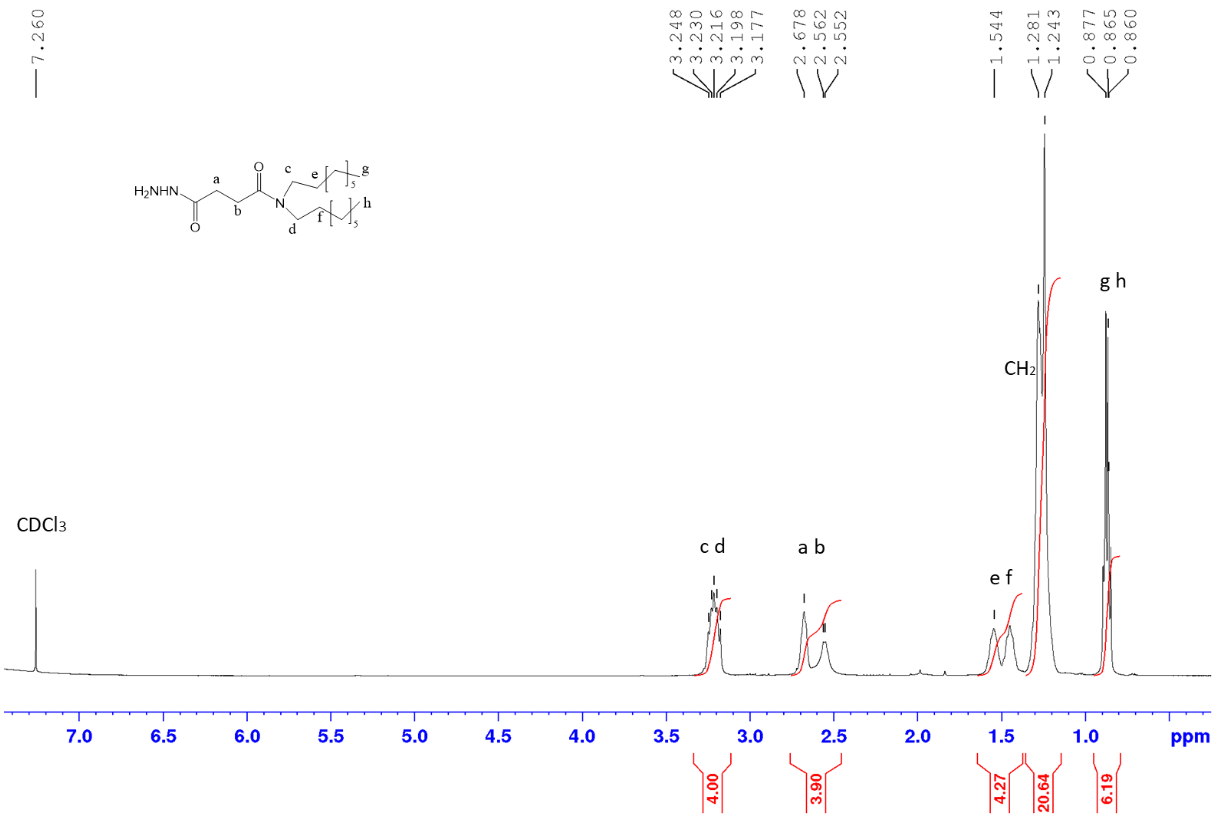

^{1}H-NMR spectrum of **3c** in $CDCl_3$.

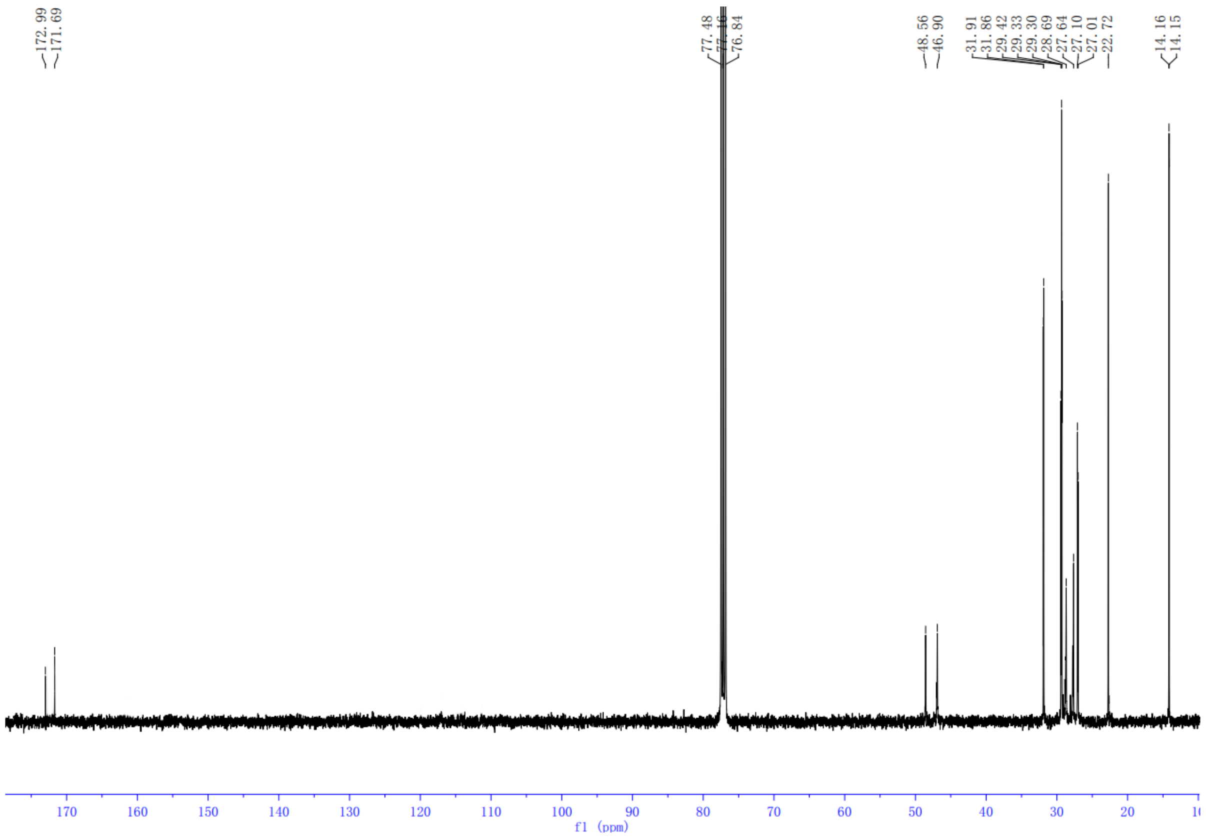

^{13}C-NMR spectrum of **3c** in $CDCl_3$.

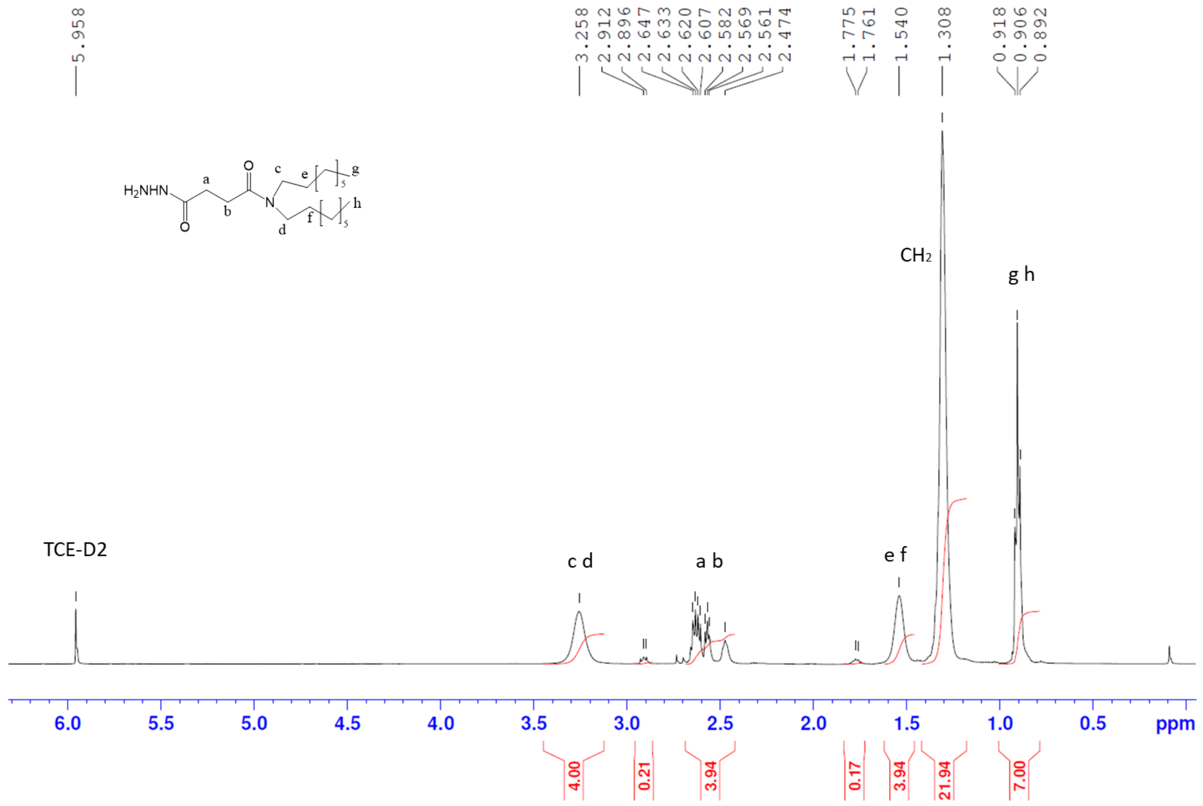

^{1}H-NMR spectrum of **3c** at high temperature (379 K) in trichloroethylene (TCE-D2). Additional minor peaks appear probably due to partial degradation of **3c** at high temperature.

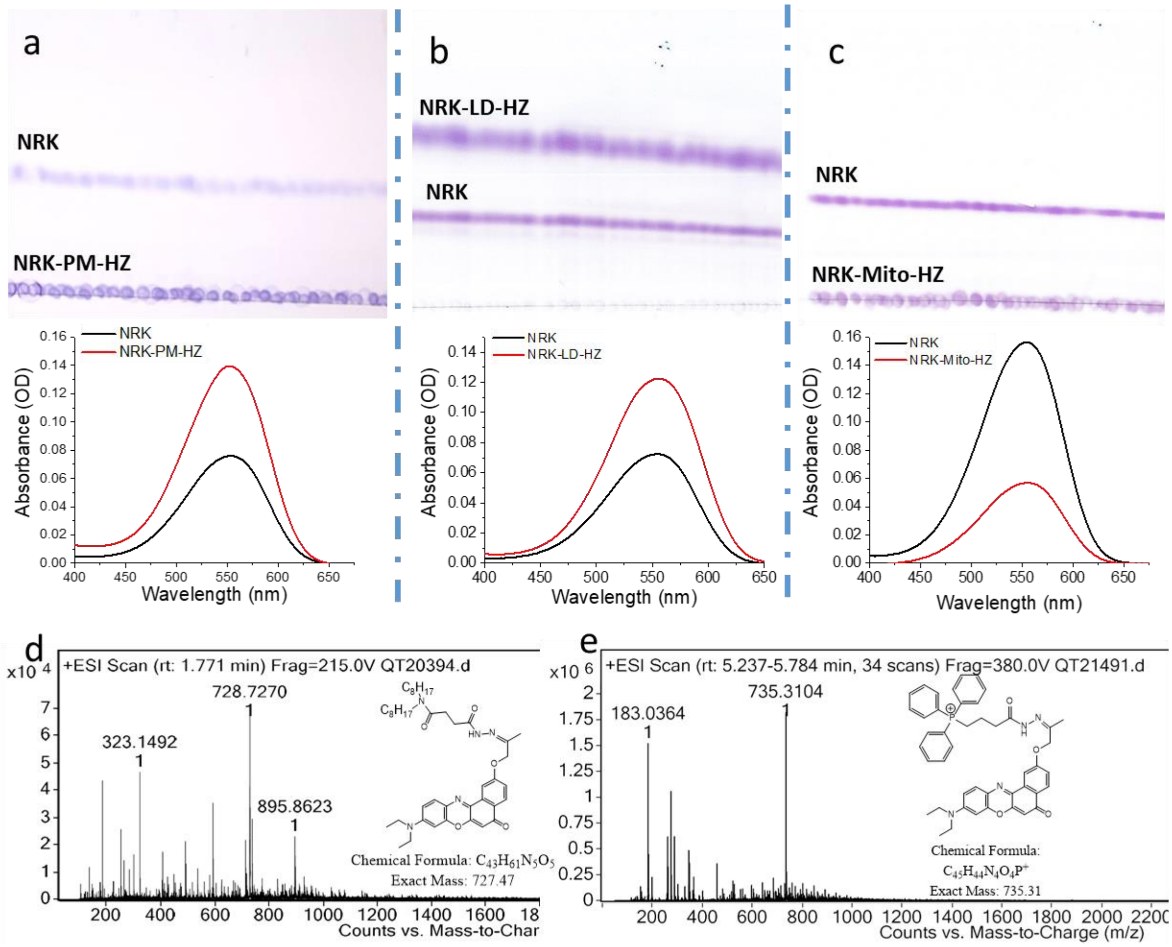

Fig. S1. (a-c) Upper panels: Photos of thin later chromatography (TLC) plates for purification (DCM:MeOH=95:5 as eluent) of the extracts from the *in situ* reactions of NRK (10 µM) with (a) PM-HZ (20 µM) in LUVs (1 mM DOPC); (b) LD-HZ (20 µM) in nanoemulsion (1 mM Labrafac oil); (c) Mito-HZ (20 µM) in LUVs (1 mM DOPC). (a-c) Upper panels: absorption spectra of NRK and the conjugates NRK-PM-HZ (a), NRK-LD-HZ (b) and NRK-Mito-HZ (c) in methanol after TLC purification. (d) Mass data for NRK-LD-HZ conjugate collected from TLC band with higher Rf (b). (e) Mass for NRK-Mito-HZ conjugate collected from TLC band with lower Rf (c).

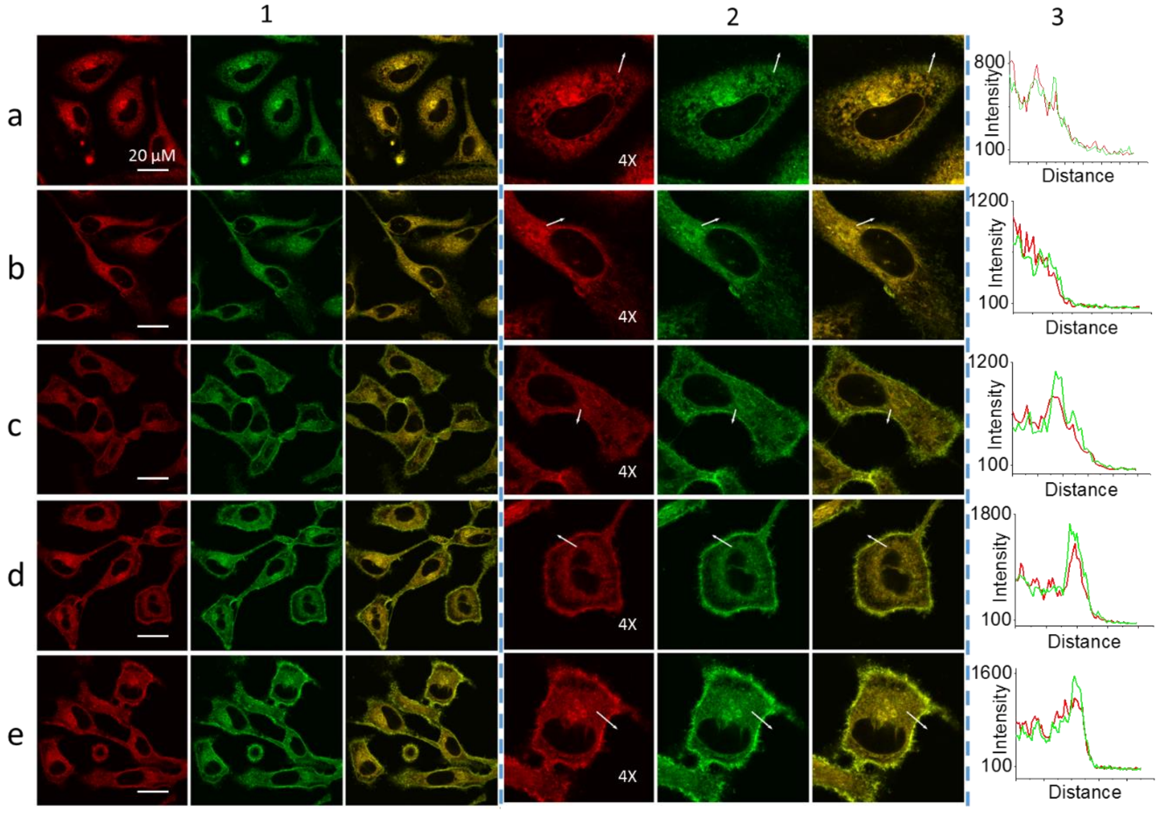

Fig. S2. Targeting NRK to plasma membrane with HZ-PM at different concentrations: 0 μM (a); 1 μM (b); 5 μM (c); 20 μM (d); 100 μM (e) following non-wash protocol. (1) Confocal images recorded in the red channel (excitation: 488 nm, emission filter: 600~670 nm) and green channel (excitation: 488nm, emission filter: 550~600 nm). (2) Corresponding zoomed (4x) images of (1). (3) Plot profile of red and green channel from white arrows indicated in images (2). Targeting agent was pre-incubated with cells followed by addition of NRK (1 μM).

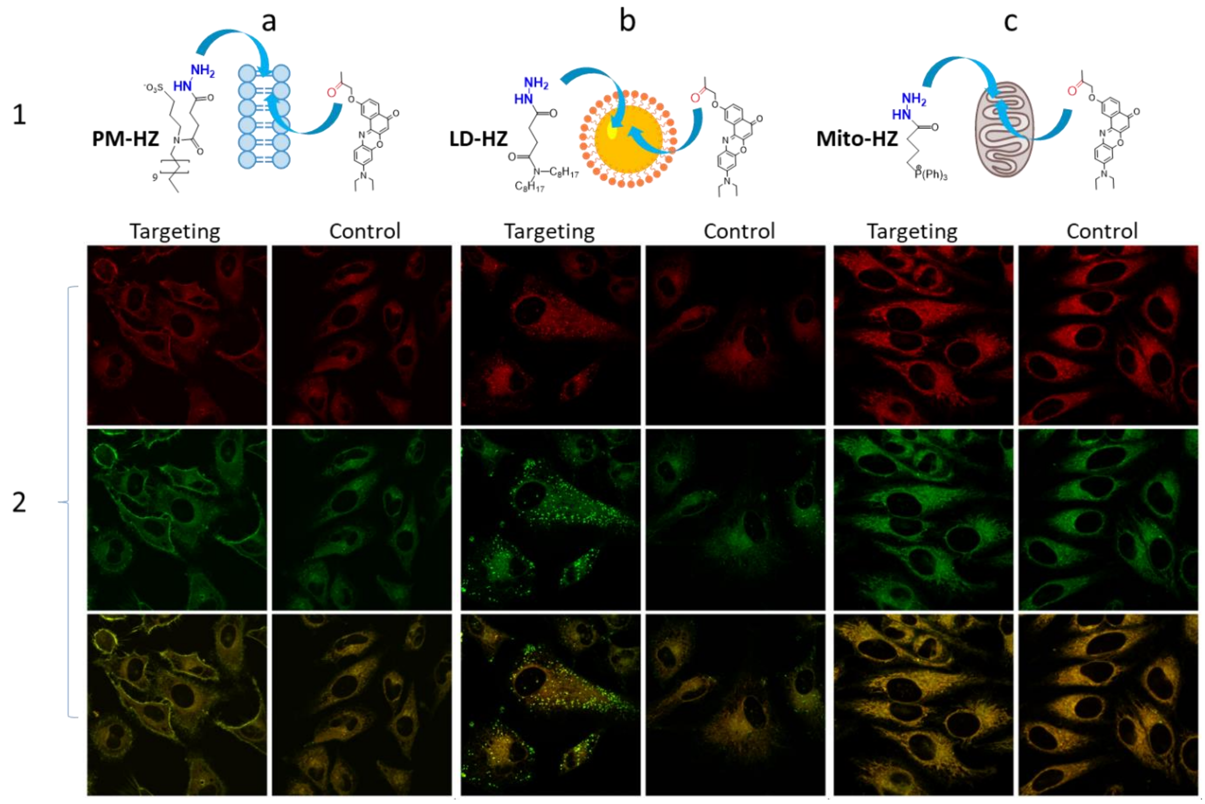

Fig. S3. Targeting NRK dye to (a) plasma membrane, (b) lipid droplets and (c) mitochondria by corresponding hydrazide ligands. (1) General scheme of targeting mechanism; (2) confocal images under condition for red channel: excitation 488 nm, emission filter of 600-670 nm; condition for green channel: excitation 488nm, emission filter of 550-600 nm. Targeting agent was pre-incubated with cells followed by washing and then addition of NRK (1 µM). In controls, NRK was used directly without the targeting agent. Zoomed images are given in Fig. 2.

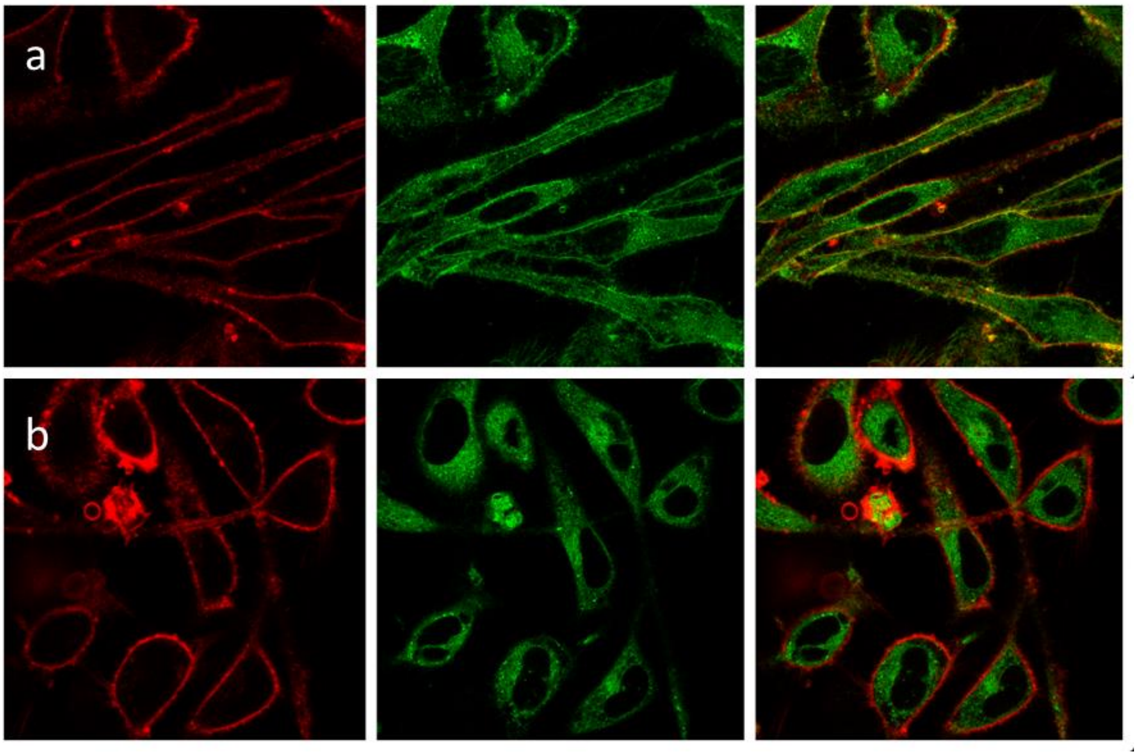

Fig. S4. Targeting NRK to plasma membranes and colocalization with membrane probe F2N12SM. Cells were pre-incubated for 10 min at 37 °C with (a) or without (b) PM-HZ (20 µM) followed by addition NRK (1 µM) and incubation for 30 min at 37 °C. Membrane staining by 400 nM F2N12SM is shown in red. Imaging conditions: for NRK, 488 nm excitation, emission filter of 550-600 nm, for F2N12SM, 405 nm excitation, emission filter of 420-480 nm.

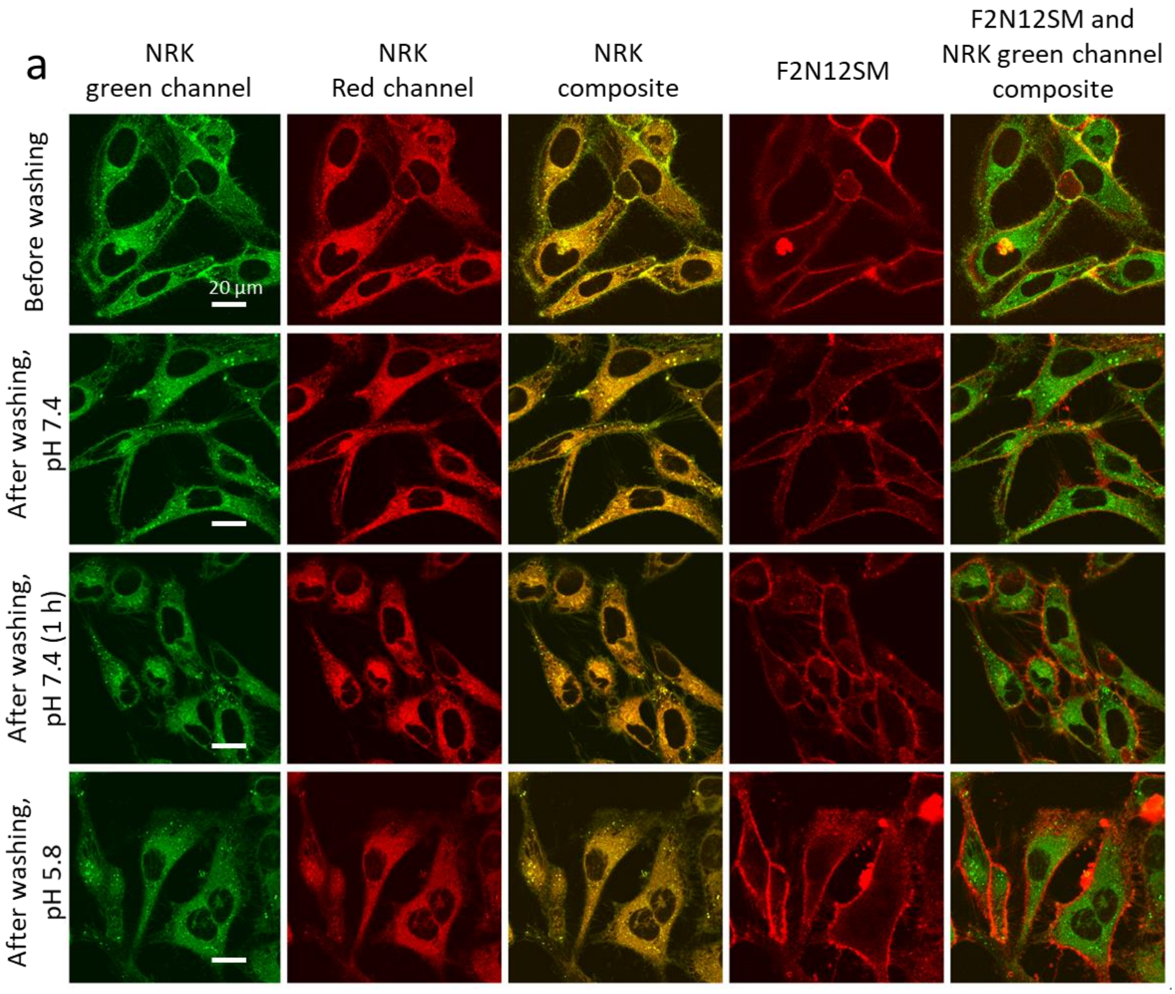
a
NRK green channel
NRK Red channel
NRK composite
F2N12SM
F2N12SM and NRK green channel composite
Before washing
After washing, pH 7.4
After washing, pH 7.4 (1 h)
After washing, pH 5.8
20 µm

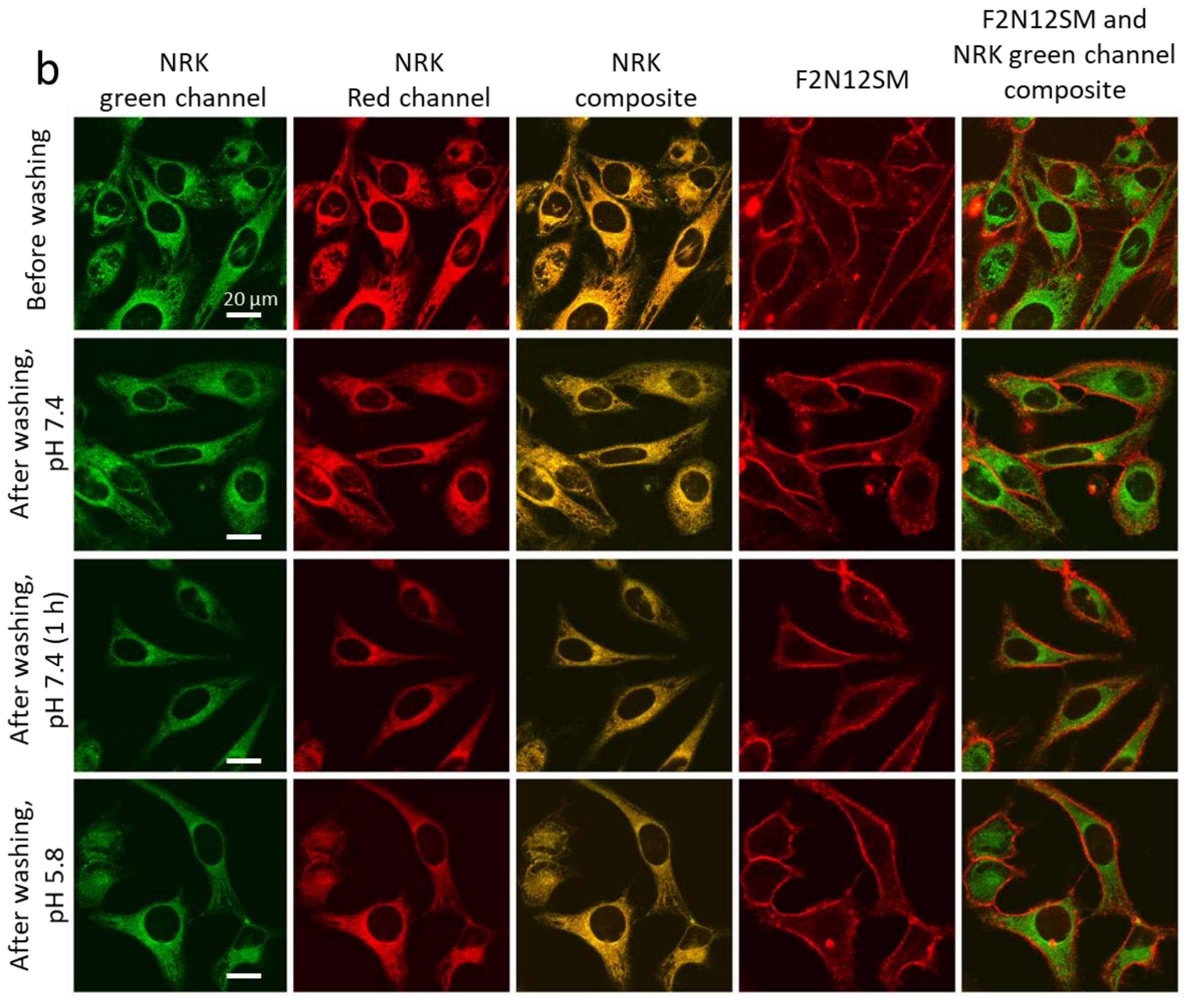

Fig. S5. Testing reversibility of NRK targeting to plasma membrane by PM-HZ in HeLa cells at neutral and low pH after washing. Plasma membranes were stained by F2N12SM. (a) NRK (1 µM) targeted to HeLa cells pretreated with PM-HZ (20 µM). (b) Controls treated with NRK (1 µM) without PM-HZ. Images were taken in Opti-MEM (pH 7.4) before washing, in Opti-MEM (pH 7.4) after washing with PBS, after long incubation (1 h) in Opti-MEM (pH 7.4) and in pH 5.8 phosphate-acetate buffer. For all four conditions before imaging, the cells were incubated for 5 min at 37 °C with plasma membrane probe F2N12SM (400 nM). Imaging conditions for the NRK red channel: excitation 488 nm, emission filter 600-670 nm; for NRK green channel: excitation 488nm, emission filter 550-600 nm; for F2N12SM: excitation 405 nm, emission filter 420-480 nm.

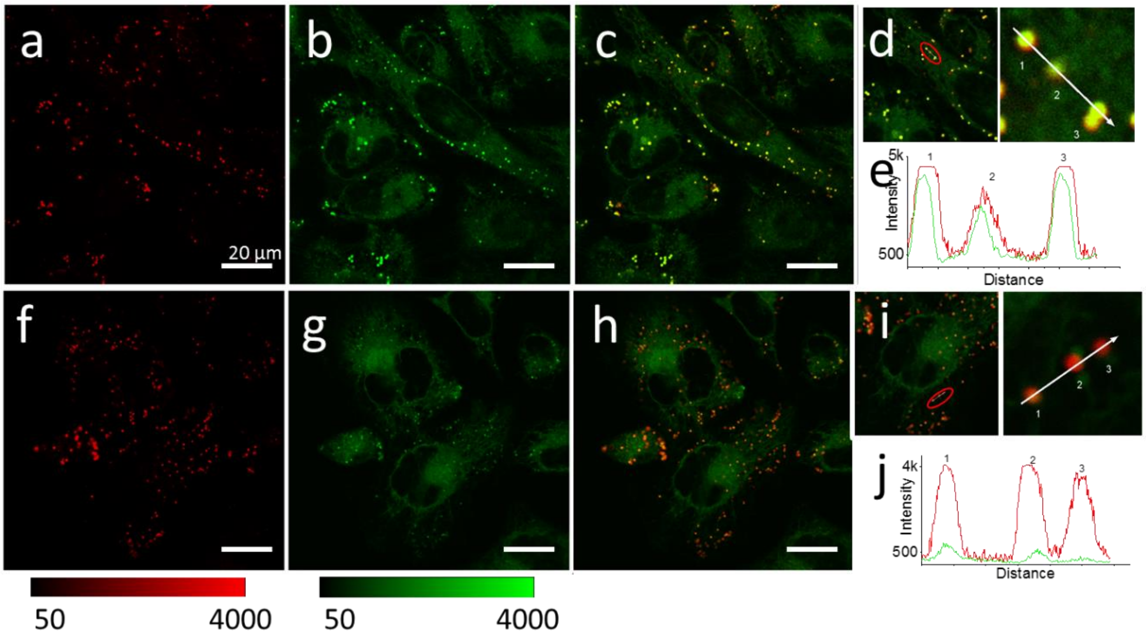

Fig. S6. Targeting of NRK (1 μM) to lipid droplets by LD-HZ (20 μM) identified by colocalization with SMCy5.5. Condition for red channel (a and f) for imaging of SMCy5.5: excitation at 644 nm, emission filter at 650-800 nm; condition for green channel (b and g): excitation at 488nm, emission filter at 550-600 nm. (c) NRK-SMCy5.5 colocalization in cells preloaded with lipid droplets targeting agent LD-HZ (overlay of a and b). (d) The selected area of (c) where the line profile analysis was performed. (d) The selected area of (c) where the line profile analysis was performed. (e) The line profile of fluorescence intensity from (d) in SMCy5.5 channel (Red) and NRK channel (Green). (h) NRK-SMCy5.5 colocalization of control cells without LD-HZ (overlay of f and g). (i) The selected area of (h) where the line profile analysis was performed. (j) The line profile of fluorescence intensity from (i) in SMCy5.5 channel (Red) and NRK channel (Green).

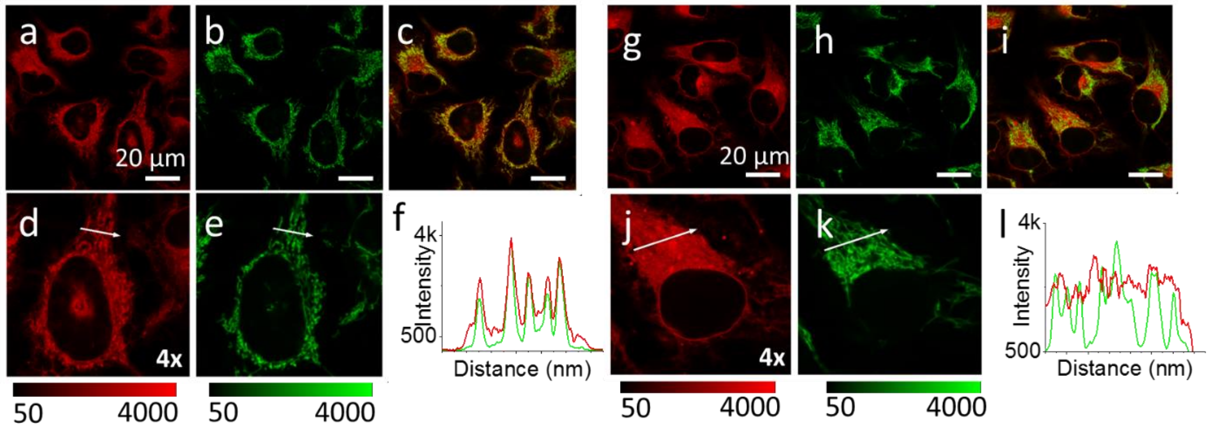

Fig. S7. Targeting NRK to mitochondria by NH-Mito identified by colocalization with MitoTracker Deep Red FM 644/665. Cells were pre-incubated for 10 min at 37 °C with (a-f) or without (g-l) Mito-HZ (20 µM), followed by cell wash with PBS and addition NRK (1 µM) and incubation for 30 min at 37 °C. Condition for red (MitoTracker Deep Red FM 644/665) channel (a,d,g,j): excitation at 635 nm, emission filter at 650-800 nm; condition for green (NRK) channel (b,e,h,k): excitation at 488 nm, emission filter at 550-600 nm. (c) NRK-MitoTracker Deep Red FM 644/665 colocalization in cells preloaded with mitochondria targeting agent Mito-HZ (overlay of a and b). (i) NRK- MitoTracker Deep Red FM 644/665 colocalization in control cells without Mito-HZ (overlay of g and h). (d, e) Selected area from (a, b) where the line profile analysis was performed; (j, k) selected area from (g, h) where the line profile analysis was performed. (f) Histogram of fluorescence intensity indicated by the white arrow in (d) and (e); (l) histogram of fluorescence intensity indicated by the white arrow in (j) and (k).

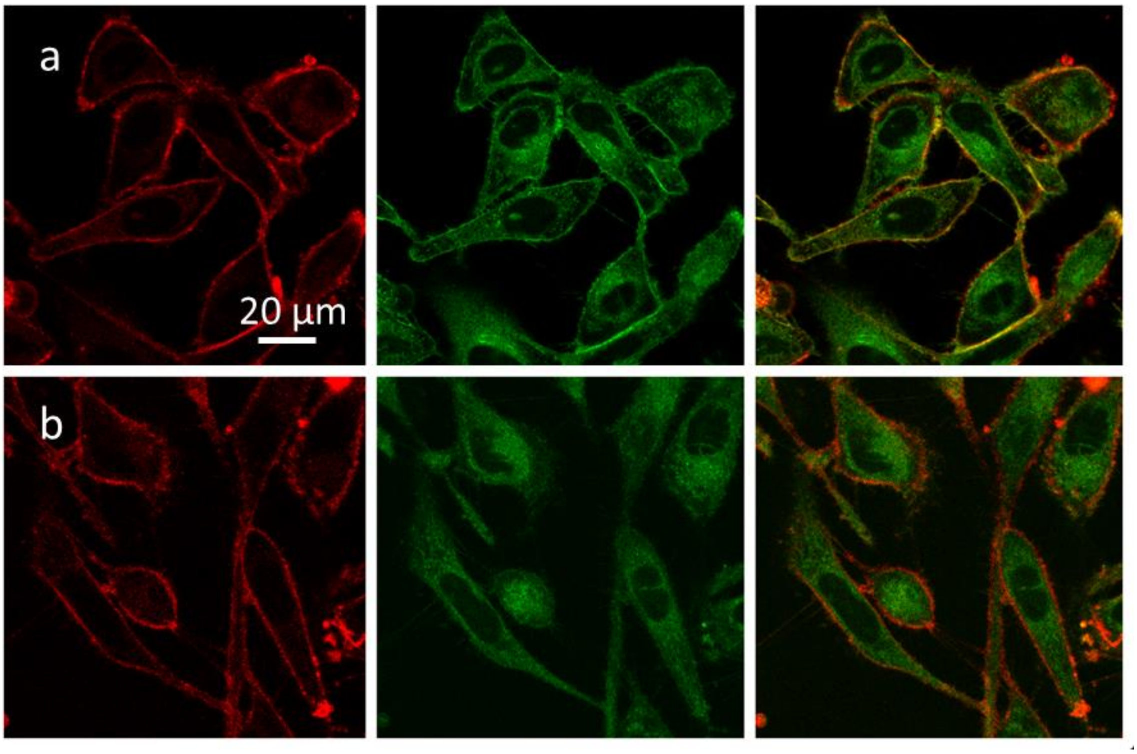

Fig. S8. Targeting doxorubicin to plasma membranes by PM-HZ and co-localization with the membrane probe F2N12SM. Confocal image of membrane co-localization of 1 µM Doxorubicine (green) by PM-HZ (20 µM) with 400 nM F2N12SM (red). (a) Targeting with PM-HZ and (b) control cells. Imaging condition: for NRK, 488 nm excitation, filter of 550-600 nm, for F2N12SM, 405 nm excitation, filter of 420 nm-480 nm.

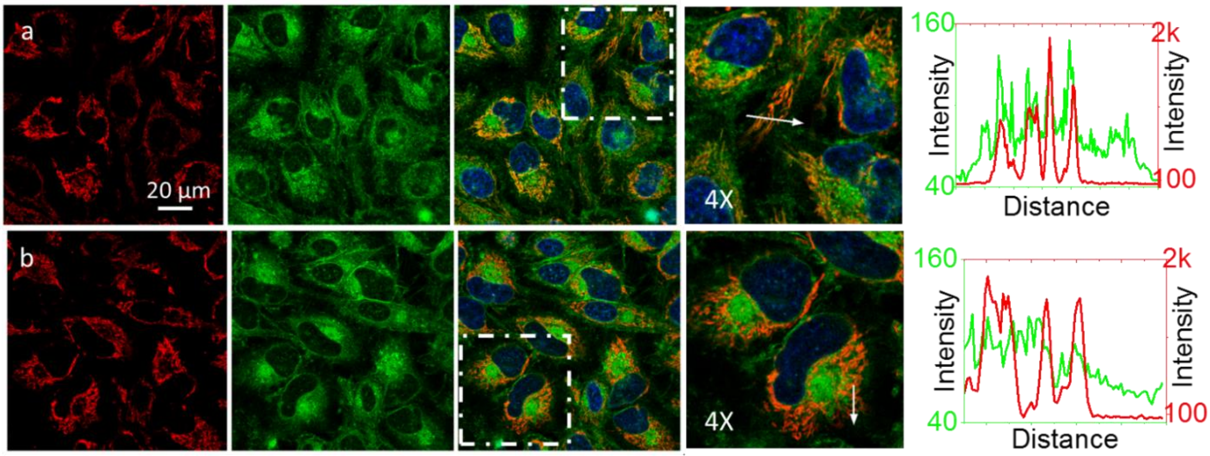

Fig. S9. Targeting doxorubicin to mitochondria by Mito-HZ. Cells were pre-incubated for 10 min at 37 °C with (a) or without (b) Mito-HZ (20 µM) followed by addition doxorubicin (1 µM) and incubation for 30 min at 37 °C. Conditions for red channel (MitoTracker Deep Red FM): excitation at 644 nm, emission filter at 650-800 nm; the green channel (doxorubicin imaging): excitation at 488 nm, emission filter at 520-650 nm; the blue channel (Hoechst 33342 imaging): excitation at 405 nm, emission filter at 420-480 nm. The line profiles (right panels) of the image in the green channel is indicated by white arrows in 4x magnified images.

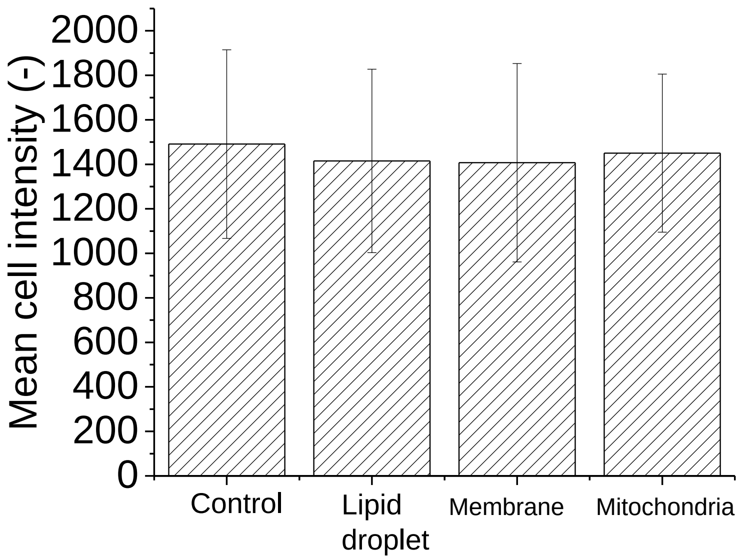

Fig. S10. Calculation of mean fluorescence intensity for cells used for the PDT experiments: control, LDs targeting (with LD-HZ), plasma membrane targeting (with PM-HZ), mitochondria targeting (with Mito-HZ). Mean fluorescence intensity was calculated for ~300 cells for each condition. Errors are standard deviation of the mean. Cells were selected by using ImageJ cell finding plugin.

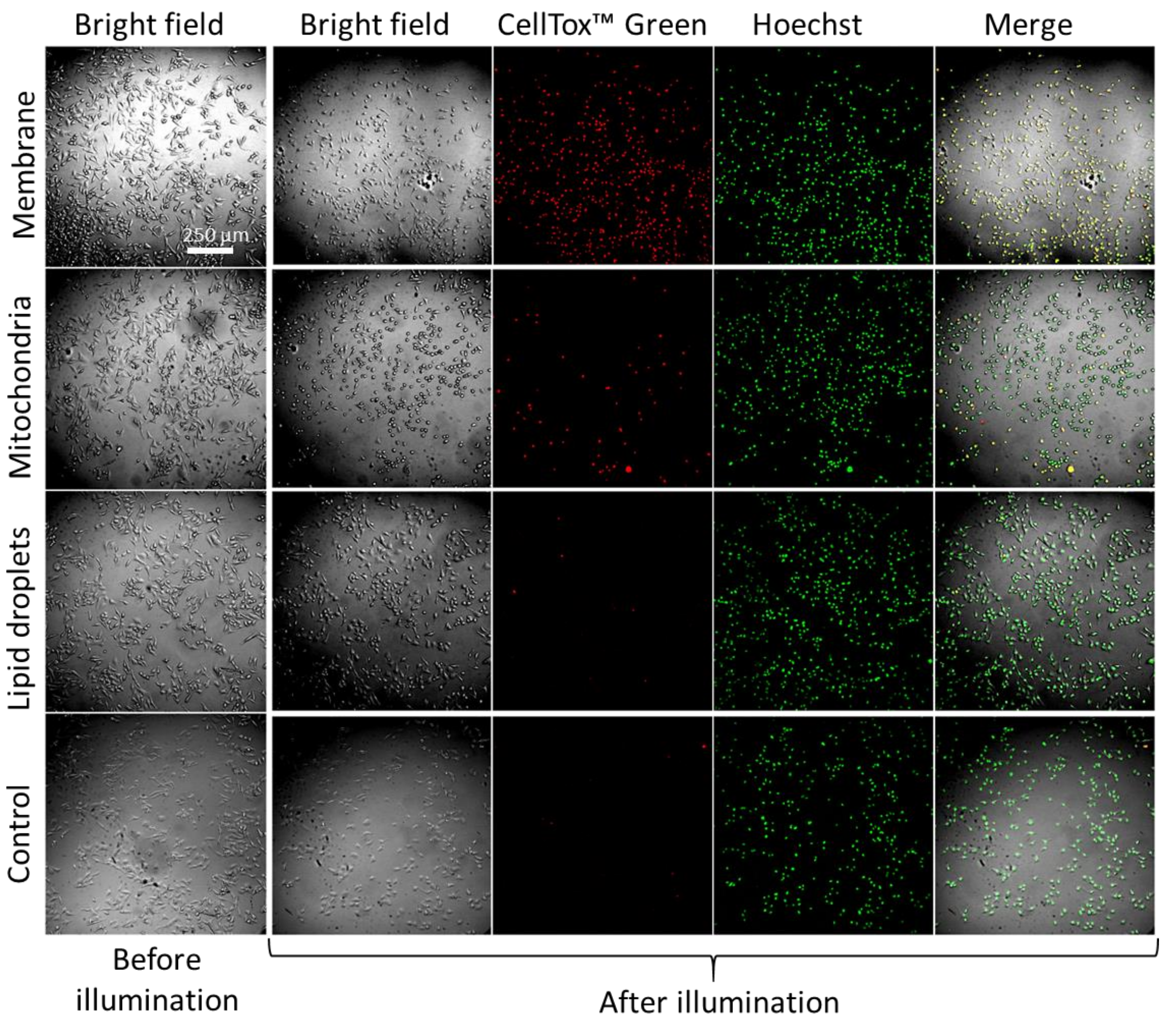

Fig. S11. Phototoxicity of NRK targeted to different organelles (plasma membranes with PM-HZ, mitochondria with Mito-HZ and lipid droplets with LD-HZ), evaluated using cell death staining agent CellTox™ Green (in Red) co-stained with nucleus staining agent Hoechst33342 (in Green). Transmission (bright field) and merged images are also shown. The cells were illuminated under microscope for 1 min at 550 nm. Images were taken after incubation for 30 min with CellTox™ Green assay.

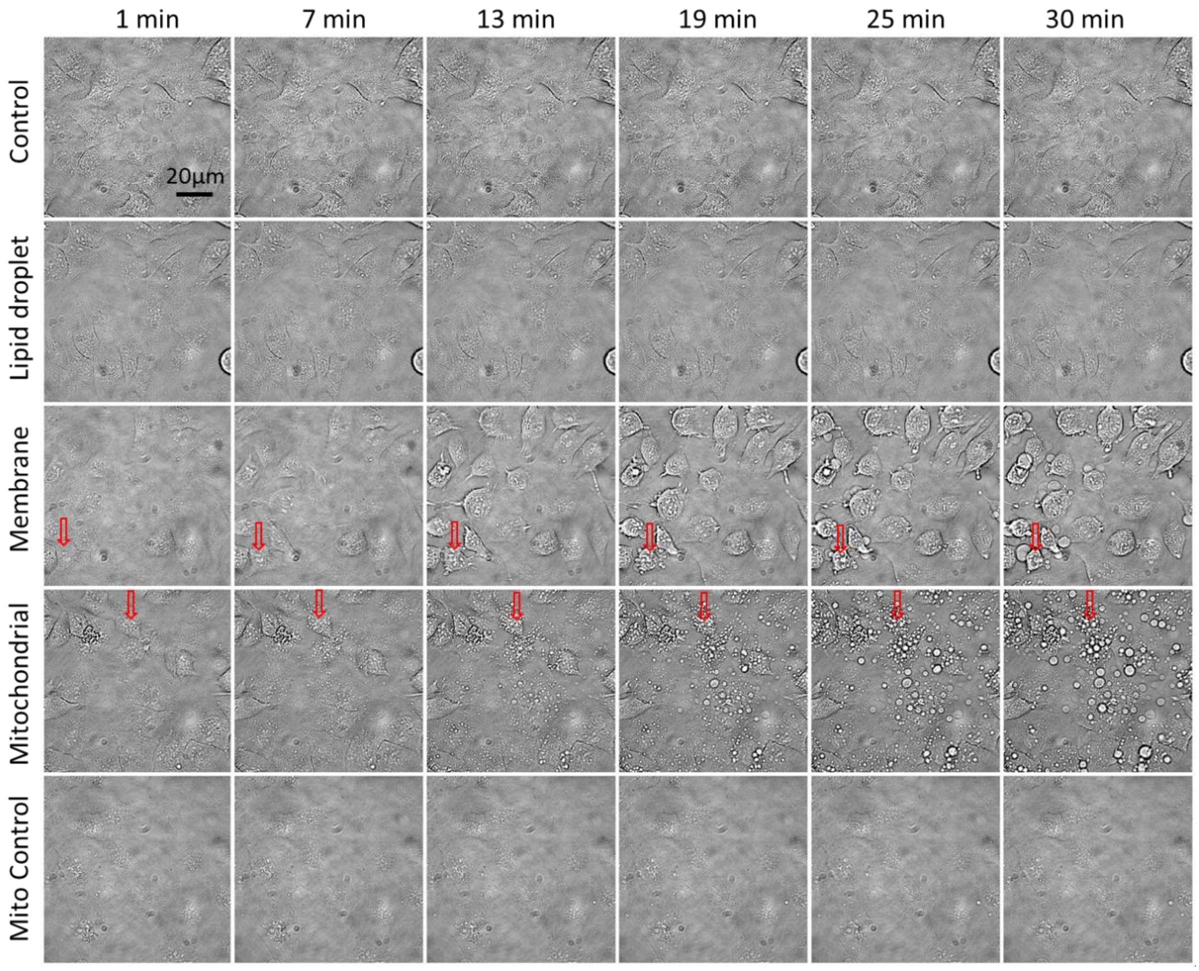

Fig. S12. Phototoxicity study based on cell morphology changes due to excitation of NRK targeted to lipid droplets, plasma membrane and mitochondria by the corresponding targeting agents. Bright-field images were taken every 1 min after excitation at 550 nm for 300 ms using high illumination power (30% for lipid droplets and plasma membrane and its control; 50 % for mitochondria and its control), images were selected for display at indicated periods of 1 min, 7 min. Red arrows show example of cells undergoing the morphological change.

Table S1. Colocalization analysis for targeting of NRK and doxorubicin (Dox) to plasma membranes, lipid droplets and mitochondria by PM-HZ, LD-HZ and Mito-HZ, respectively.[a]

Sample / Parameter	Plasma membranes				Lipid droplets				Mitochondria			
	NRK		Dox		NRK		Dox		NRK		Dox	
	Control	Target	Control	Target	Control	Target	Control	Target	Control	Target	Control	Target
Intensity ratio (Organelle/cell)	0.88±0.11	2.06±0.35	1.02±0.23	1.50±0.23	1.67±0.32	4.46±0.60	1.98±0.048	8.45±0.25	1.58±0.12	1.73±0.39	1.46 ±0.12	1.52±0.085
Pearson's correlation coefficient	0.20±0.038	0.55±0.01	0.21±0.042	0.52±0.068	0.52±0.026	0.68±0.041	0.16±0.034	0.58±0.068	0.65±0.036	0.75±0.028	0.34±0.018	0.38±0.026
Mander's correlation coefficient	0.13±0.033	0.42±0.001	0.21±0.042	0.35±0.074	0.26±0.068	0.77±0.081	0.04±0.029	0.57±0.068	0.54±0.034	0.44±0.11	0.31±0.071	0.30±0.082

[a] 15 cells were analysed per condition.

Table S2. Colocalization analysis for reversibility of NRK labelling of plasma membranes with PM-HZ with respect to reference dye F2N12SM.[a]

Experiment / Parameter	Targeting group				Control group			
	Before washing	After washing, pH 7.4	After washing, pH 7.4 (1 h)	After washing, pH 5.8	Before washing	After washing, pH 7.4	After washing, pH 7.4 (1 h)	After washing, pH 5.8
Intensity ratio (Organelle/cell)	1.71±0.12	1.28±0.10	1.10±0.021	0.85±0.02	0.86±0.010	0.80±0.11	1.10±0.019	0.85±0.016
Pearson's correlation coefficient	0.49±0.045	0.32±0.075	0.32±0.064	0.22±0.030	0.14±0.11	0.16±0.072	0.25±0.064	0.19±0.023
Mander's correlation efficiency	0.55±0.056	0.53±0.065	0.42±0.028	0.36±0.037	0.21±0.033	0.18±0.022	0.30±0.053	0.19±0.056

[a] 15 cells were analysed per condition.

References

1. F. Liu, Y. Niko, R. Bouchaala, L. Mercier, O. Lefebvre, B. Andreiuk, T. Vandamme, J. G. Goetz, N. Anton and A. Klymchenko, *Angew. Chem. Int. Ed.*, 2021, **60**, 6573-6580.
2. M. Collot, T. K. Fam, P. Ashokkumar, O. Faklaris, T. Galli, L. Danglot and A. S. Klymchenko, *J. Am. Chem. Soc.*, 2018, **140**, 5401-5411.
3. R. Kreder, S. Oncul, O. A. Kucherak, K. A. Pyrshev, E. Real, Y. Mély and A. S. Klymchenko, *RSC Adv.*, 2015, **5**, 22899-22905.
4. D. I. Danylchuk, S. Moon, K. Xu and A. S. Klymchenko, *Angew. Chem. Int. Ed.*, 2019, **58**, 14920-14924.
